# Faster than expected Rubisco deactivation in shade reduces cowpea photosynthetic potential in variable light conditions

**DOI:** 10.1038/s41477-021-01068-9

**Published:** 2022-01-20

**Authors:** Samuel H. Taylor, Emmanuel Gonzalez-Escobar, Rhiannon Page, Martin A. J. Parry, Stephen P. Long, Elizabete Carmo-Silva

**Affiliations:** 1 Lancaster Environment Centre, Lancaster University, Lancaster, UK; 2 Departments of Plant Biology and of Crop Sciences, Carl R. Woese Institute of Genomic Biology, University of Illinois, Urbana, IL USA

**Keywords:** Plant physiology, C3 photosynthesis, Rubisco, Natural variation in plants, Light responses

## Abstract

Cowpea is the major source of vegetable protein for rural populations in sub-Saharan Africa and average yields are not keeping pace with population growth. Each day, crop leaves experience many shade events and the speed of photosynthetic adjustment to this dynamic environment strongly affects daily carbon gain. Rubisco activity is particularly important because it depends on the speed and extent of deactivation in shade and recovers slowly on return to sun. Here, direct biochemical measurements showed a much faster rate of Rubisco deactivation in cowpea than prior estimates inferred from dynamics of leaf gas exchange in other species^1–3^. Shade-induced deactivation was driven by decarbamylation, and half-times for both deactivation in shade and activation in saturating light were shorter than estimates from gas exchange (≤53% and 79%, respectively). Incorporating these half-times into a model of diurnal canopy photosynthesis predicted a 21% diurnal loss of productivity and suggests slowing Rubisco deactivation during shade is an unexploited opportunity for improving crop productivity.

## Abstract (web summary)

The crucial enzyme for photosynthesis, Rubisco, is deactivated during periods of shade and slowly recovers when illuminated. In fluctuating light conditions crop productivity could be substantially increased by slowing Rubisco deactivation during shade.

## Main

Some 240 million people in sub-Saharan Africa are malnourished and this has been steadily worsening over the past 6 years. Regional improvement in food production lags behind that of most of the world, yet population growth is high, suggesting that numbers of seriously malnourished people will continue to increase^4^. Cowpea (*Vigna unguiculata* (L.) Walp.) is the most important plant protein source for rural sub-Saharan Africa but its productivity has increased little over the past decade^4–6^.

Despite being the source of all plant matter, improvement of photosynthesis is a largely unexploited opportunity that has only recently been implemented to drive large increases in rates of biomass production in tobacco and rice^7,8^. Although focus has been on improving steady-state light-saturated rates of photosynthesis, evidence suggests that major gains in plant productivity could be obtained by improving adjustment to the continual light fluctuations that occur within crop canopies in the field. By transgenically upregulating genes that affect the speed with which photosynthetic efficiency adjusts to sun–shade transitions, productivity of field-grown tobacco increased 14–20% (ref. ^9^).

Canopy modelling using measured rates of photosynthetic induction during shade–sun transitions suggests a means to gains of similar magnitude^1,3,10^. A key factor controlling speed of induction is the activity of the ATP-dependent metabolic repair chaperone, Rubisco activase (Rca; see also Supplementary Table 1 for abbreviations). The assumed mechanism of Rubisco (ribulose-1,5-bisphosphate carboxylase–oxygenase) activation is that Rca removes tightly bound inhibitory sugar-phosphates from catalytic sites, allowing carbamylation; that is, reversible binding of CO_2_ and Mg^2+^, and in turn carboxylation or oxygenation of ribulose-1,5-bisphosphate (RuBP)^11^. Establishing the potential impact of Rubisco activation on photosynthetic productivity requires modelling the response of Rubisco activity to realistic within-crop canopy light regimes^1–3^.

Shade is an obvious limit on photosynthesis in forest and understorey plants^12^. Within dense short-stature crop canopies like soybean, wheat and cowpea, most leaves also experience many transitions between sun and shade^3,13,14^. Throughout a day, light reaching chloroplasts steps-up or steps-down by 90% within a second^3^. In shade, biochemical adjustments improve the efficiency with which chloroplasts use absorbed light^9^ but the light-dependent supply of RuBP is insufficient to saturate Rubisco catalytic sites, allowing decarbamylation and/or sugar-phosphate inhibition to decrease Rubisco activity^15–17^. Following shade–sun transitions, Rubisco activation is among the slowest responding of the biochemical processes that tune photosynthetic capacity to match incoming light^18,19^.

Shade–sun transitions are initially followed by RuBP regeneration driven, fast increases in photosynthesis, quickly superseded by prolonged, slower recovery driven by Rubisco activation^19^. Rates of increase in CO_2_ assimilation during induction have therefore been used to infer rates of Rubisco activation^16,20^ and have shown diversity that could be exploited to improve crop productivity^2,21,22^. By contrast, the rate of Rubisco deactivation following sun–shade transitions has never been characterized in a grain crop using both in vitro assays and gas exchange. A foundational study using both methods with spinach^16^ found long deactivation half-times of >1,440 s; however, subsequent gas exchange measurements estimated only 606 s for the same species^23^. Furthermore, basil and impatiens showed faster Rubisco deactivation on the basis of in vitro biochemistry than gas exchange^17^. Parameterization of Rubisco deactivation therefore remains a key uncertainty in addressing impacts of Rubisco activation on crop productivity^2^.

The match between in vivo (leaf gas exchange) and in vitro (Rubisco activity) measurements, and the potential gain in diurnal photosynthesis achievable by adjusting the response of Rubisco activity to shade, were evaluated in cowpea. Activation state during sun–shade–sun transitions was measured using an optimized in vitro leaf-disc approach^24,25^. A uniform light regime was imposed with balanced spectrum LED lighting and temperature control (Supplementary Fig. 1) and light responses of Rubisco activation state were obtained under steady-state and with temporal resolution down to 15 s during sun–shade–sun sequences. Results were used to update a diurnal model that combines a light regime for a legume canopy^14^; half-times (*τ*) for the Rubisco activation state (*S*) response to step changes in light^16,26^; and net CO_2_ assimilation (*A*) based on steady-state light-response curves^1^. In parallel, the model was parameterized using gas exchange-based *τ* for the maximum rate of carboxylation by Rubisco (*V*_c,max_). To indicate potential for impacts of breeding on Rubisco activity, two *V. unguiculata* breeding lines (IT86D-1010 and IT82E-16), a sexually compatible wild relative *Vigna* sp. Savi (TVNu-1948) and a more distantly related perennial *V. adenantha* (L.) were compared.

**Fig. 1:**
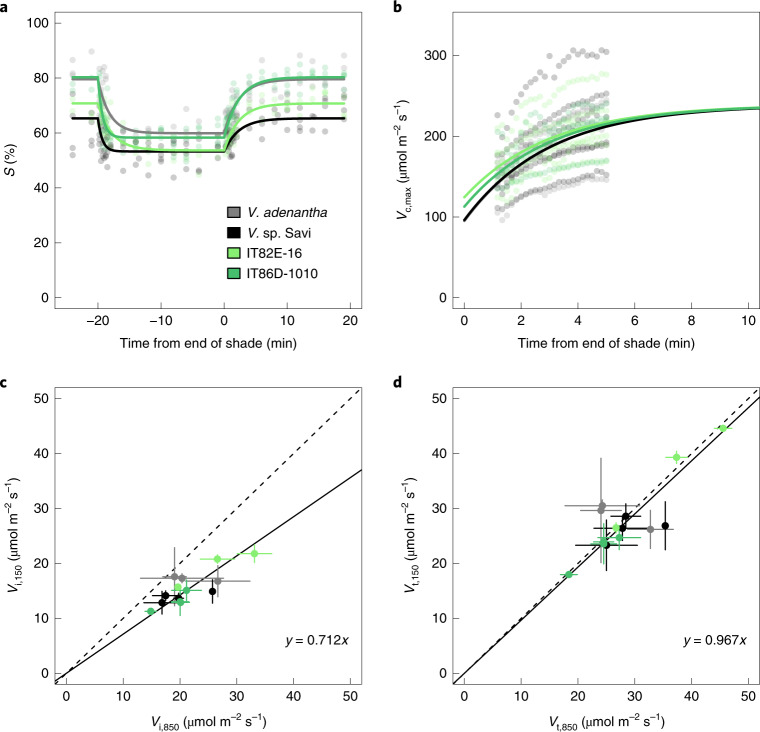
Rubisco activation responses to sun–shade–sun at 30 °C. **a**, Rubisco activation state measured in vitro (*S*; individuals per accession: *n* = 3, IT82E-16 and *V. adenantha*; *n* = 4, IT86D-1010 and *Vigna* sp. Savi). **b**, Maximum Rubisco carboxylation rate (*V*_c,max_) modelled from gas exchange measurements (individuals per accession: *n* = 4, IT86D-1010 and *V. adenantha*; *n* = 6, IT82E-16 and *Vigna* sp. Savi). Points show time series for individuals, lines are fixed effects predictions from nonlinear mixed-effects models that accounted for among-individual variation; in **b**, the model is extrapolated beyond the period 1–5 min after shade when *V*_c,max_ limited net CO_2_ assimilation. The response of components of Rubisco activation state are shown using equivalence plots for steady-state. **c**,**d**, Initial (*V*_i_) (**c**) and total (*V*_t_) (**d**) Rubisco activity in sun (850 μmol m^−2^ s^−1^, after recovery of *S*) and shade (150 μmol m^−2^ s^−1^, immediately preceding the end of shade). Means and s.d. are shown for individual plants (two to three technical replicates; individuals per accession: *n* = 3, IT82E-16 and *V. adenantha*; *n* = 4, IT86D-1010 and *Vigna* sp. Savi), along with a 1:1 reference (dashed line) and regression of *y* = *ax* for the means (solid line, *n* = 14 individuals). *V*_i_, without pre-incubation with effectors Mg^2+^ and CO_2_, responded significantly to shade (*a* = 0.712, 95% CI 0.65, 0.77) and *V*_t_ did not (*a* = 0.967, 95% CI 0.89, 1.04). Four *Vigna* accessions were characterized, including two cowpea breeding lines (IT86D-1010 and IT82E-16) and two wild species (*V. adenantha* and *Vigna* sp. Savi). In both biochemistry and leaf gas exchange experiments, material was brought to steady-state photosynthesis in saturating light, then shaded for 20 min before returning in a single step to the initial light level.

For all accessions, *S* saturated at a photosynthetic photon flux density (PPFD) of ~600 μmol m^−2^ s^−1^ (Supplementary Fig. 2). Sun–shade–sun sequences were simulated using 850 μmol m^−2^ s^−1^ (sun) and 150 μmol m^−2^ s^−1^ (shade) (Fig. 1a). In shade, *S* decreased with a half-time (*τ*_d,S_) of 42–134 s, depending on the accession (*F*_3,374_ = 13.2, *P* = 3.2 × 10^–8^; Table 1). Deactivation of Rubisco in *Vigna* sp. Savi and IT86D-1010 was so rapid that *τ*_d,S_ was not statistically resolvable from 0; by contrast, *τ*_d,S_ for *V. adenantha* and IT82E-16 was ~120 s (Table 1). Thus, *τ*_d,S_ was as different within cowpea as between *Vigna* species. In shade, *S* decreased by 18–28% and accessions with high *S* in sun also showed higher *S* in shade. *S* was greater in *V. adenantha* and IT86D-1010 than in the other two accessions (*F*_3,374_ = 14.9, *P* < 3.4 × 10^–9^; Table 1), so there was no clear association between *S* and *τ*_d,S_. For Rubisco activation, the half-time of induction (*τ*_a,S_) did not differ among the accessions (*F*_3,371_ = 1.56, *P* = 0.2) and was 144 s (Table 1). Estimates of *τ*_a_ for other crops derived from gas exchange range from ~100 to 350 s (refs. ^1,2,17,20,21,27^) and decrease at higher assay temperatures as used here^27^.

**Table 1:** Photosynthetic induction parameters at 30 °C for four *Vigna* accessions

	*S*_H_	*S*_L_	*τ*_d,S_^a^	*τ*_a,S_^a^
	(%)	(%)	(s)	(s)
*V. adenantha*	80 ± 2.9^A^	59 ± 3.4^A^	108 ± 47^A^	144 ± 27
*V.* sp. Savi	65 ± 3.8^B^	53 ± 4.3^B^	42 ± 48^B^	
IT82E-16	71 ± 4.0^C^	54 ± 4.7^BC^	132 ± 70^A^	
IT86D-1010	80 ± 3.8^A^	58 ± 4.3^AC^	42 ± 51^B^	
	***V***_**c,max,H**_	***V***_**c,max,L**_	***τ***_**d,V**_^a,b^	***τ***_**a,V**_^a^
	**(μmol** **m**^**−2**^ **s**^**−1**^**)**	**(μmol** **m**^**−2**^ **s**^**−1**^**)**	**(s)**	**(s)**
*V. adenantha*	239 ± 17.6	95 ± 17.1^A^	241	180 ± 24
*V.* sp. Savi		96 ± 19.7^A^	242	
IT82E-16		124 ± 20.4^B^	253	
IT86D-1010		113 ± 22.2^AB^	248	
	***φ***^a^	***A***_**sat**_^a^	***θ***^a^	***R***_**d**_^a^
	**–**	**(μmol** **m**^**−2**^ **s**^**−1**^**)**	**–**	**(μmol** **m**^**−2**^ **s**^**−1**^**)**
*V. adenantha*	0.059 ± 0.0050^AB^	32 ± 3.8^A^	0.83 ± 0.043^A^	1.52 ± 0.419^A^
*V.* sp. Savi	0.058 ± 0.0050^B^	34 ± 3.8^AB^	0.8 ± 0.043^AB^	1.58 ± 0.419^A^
IT82E-16	0.063 ± 0.0035^A^	39 ± 2.7^C^	0.78 ± 0.031^B^	2.17 ± 0.296^B^
IT86D-1010	0.063 ± 0.0050^A^	36 ± 3.8^BC^	0.77 ± 0.044^B^	1.85 ± 0.420^AB^

The photosynthetic induction parameters are based on Rubisco activation state measured in vitro (*S*) or maximum Rubisco carboxylation rate from gas exchange (*V*_c,max_; final steady-state at high light (subscript H), initial during shade (subscript L)), their characteristic half-times during activation (*τ*_a_) and deactivation (*τ*_d_) and parameters of the A/PPFD curves used in diurnal modelling (*φ*, initial slope at low PPFD; *A*_sat_, asymptotic rate at high PPFD; *θ*, a curvature parameter; and *R*_d_, day respiration). Values are means ± 95% CI for fixed effects. Genotype-level fixed effects were included in models only where significant.

The fit of models at the level of individual plants is shown in Supplementary Figs. 9, 10 and 11; parameter values from an alternative individual-by-individual model are shown in Supplementary Tables 2 and 3. Different capital superscript letters indicate non-overlap of 95% CI.

^a^Used in diurnal modelling.

^b^Calculated from mean *V*_c,max,H_ and *V*_c,max,L_ (equation (7)): not modelled using mixed effects.

The behaviour of *S* and *V*_c,max_ differed. Unlike *S*, *V*_c,max_ of the four accessions was similar in high light (coefficient contrasts *P* ≥ 0.64). While confidence intervals (CIs, 95%) did indicate significantly lower shade values in wild *Vigna* compared with IT82E-16, between-accessions patterns of difference between *S* and *V*_c,max_ did not correspond (Table 1 and Fig. 1b). Such correspondence is not expected because, in addition to *S*, *V*_c,max_ depends on Rubisco amount and catalytic properties. The apparently larger decrease in Rubisco activity in shade based on *V*_c,max_ (48–60%; Table 1 and Fig. 1b) compared to *S* was linked with longer *τ*_V_ than *τ*_S_. Similarly, the half-time for increasing *V*_c,max_ (*τ*_a,V_) was 26% longer than *τ*_a,S_ (*P* ≤ 0.05 on the basis of 95% CIs; Table 1). Half-times for decreasing *V*_c,max_ (*τ*_d,V_) were calculated dependent on *V*_c,max,H_ and *V*_c,max,L_ (equation (7)) so CIs were not estimated for *τ*_d,V_ but at 241–253 s they were 1.9–5.8 × *τ*_d,S_, depending on accession and were longer than the upper 95% CIs for *τ*_d,S_ (Table 1). Because estimates of *τ*_d,V_ assumed that after 20 min shade *V*_c,max_ was within 1 μmol m^−2^ s^−1^ of the asymptote (*V*_c,max,L_) (equations (6), (7)) and *S* stabilized faster than this, *τ*_d,V_ overestimated *τ*_d_ (Fig. 1a).

Initial activity of Rubisco in shaded leaf discs stabilized at ~70% of the value at high light (Fig. 1c). Assays of total activity, following carbamylation of catalytic sites free of sugar-phosphates, showed no response to PPFD (Fig. 1d). Carbamylation relies on stromal pH, [CO_2_] and [Mg^2+^] and the availability of inhibitor-free Rubisco catalytic sites depends on [RuBP] and Rca activity^28^. In shade, *A* diminishes and stomata will open at low [CO_2_], so CO_2_ seems unlikely to be limiting. The relative importance of stromal pH and [Mg^2+^] as companions to Rca activity controlling Rubisco carbamylation in shade remain to be established but in model plant species expressing varying amounts^20^ and isoforms^25^ of Rca, slowing deactivation and speeding induction by Rca-mediated maintenance of Rubisco activity shows promise as a strategy to enhance productivity.

**Fig. 2:**
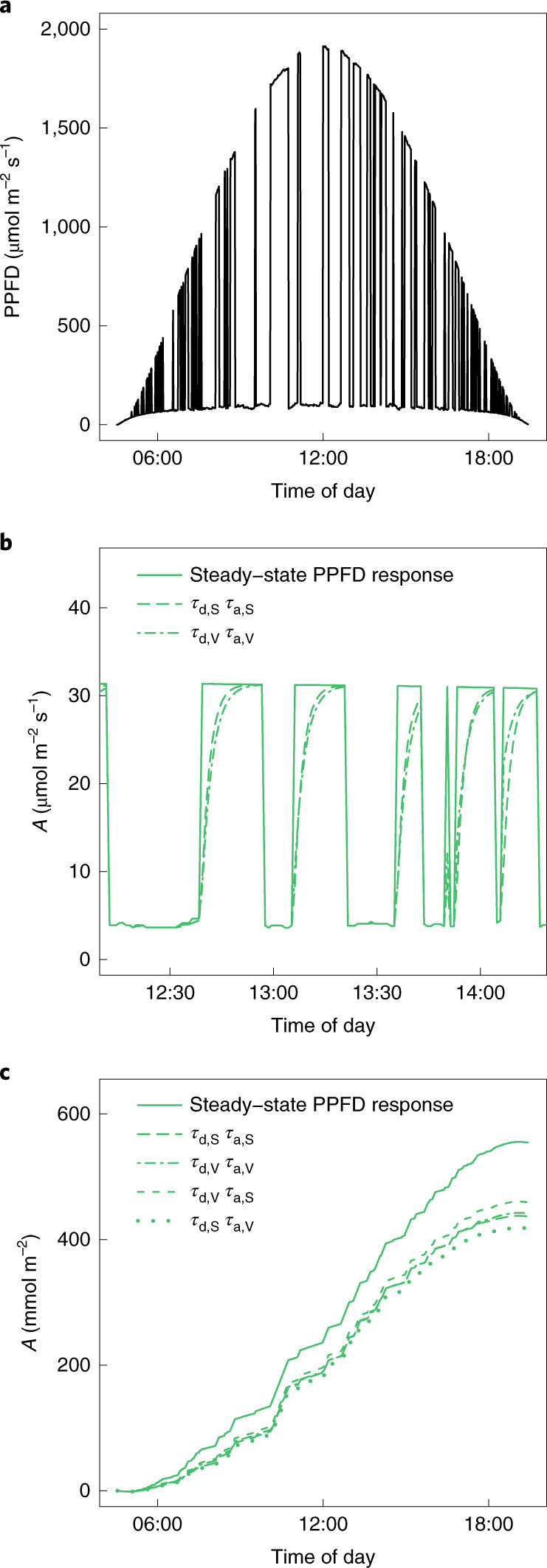
Modelling the diurnal impacts of slow changes in Rubisco activity at 30 °C. **a**, Light regime for a chloroplast in a second-layer legume canopy^14^. **b**, Mid-day segment of diurnal time series for the cowpea line IT86D-1010. Lags relative to tracking of a steady-state PPFD response are shown for modelling based on Rubisco activity (*τ*_d,S_, *τ*_a,S_) compared with gas exchange measurements (*τ*_d,V_, *τ*_a,V_); note the impact of shade duration on differences between the models. **c**, Cumulative assimilation during the diurnal period for the scenarios in **b**, alongside models simulating the effect of slower deactivation (*τ*_d,V_, *τ*_a,S_) and slower activation (*τ*_d,S_, *τ*_a,V_) of Rubisco.

Important diurnal impacts of Rubisco activation previously reported for wheat^1^ were based on in vivo estimates of Rubisco activity (*τ*_d,V_ and *τ*_a,V_). Here, Rubisco deactivation and activation half-times determined both in vivo and in vitro (*τ*_d,S_ and *τ*_a,S_), were used to model photosynthetic adjustment to diurnal light fluctuations within the second layer of a canopy (Fig. 2). Both in vivo and in vitro approaches predicted foregone assimilation linked with Rubisco activation (*A*_f_) matching the 21% of diurnal photosynthetic potential (*A*_Q_; Table 2 and Fig. 2c) predicted for wheat^1^. Significant differences in light-response characteristics between the four *Vigna* accessions (Table 1) had little impact on diurnal photosynthesis (*A*_Q_: coefficient of variation, 3.9%; Table 2) relative to the ~21% reduction linked with Rubisco regulation (*A*_f_; Table 2). Noting that *τ*_d,V_ represents an upper limit for reasons given above, and that *τ*_V_ were longer than *τ*_S_, we used these values reciprocally to establish the potential impact of modifying *τ*_d_ and *τ*_a_. Both slowing-down deactivation (*τ*_d,V_ + *τ*_a,S_ versus *τ*_d,S_ + *τ*_a,S_) and speeding-up activation (*τ*_d,V_ + *τ*_a,S_ versus *τ*_d,V_ + *τ*_a,V_) significantly decreased *A*_f_ to 17% (on the basis of 95% CIs; Table 2). Similarly, slowing activation following shade (*τ*_d,S_ + *τ*_a,V_ versus *τ*_d,S_ + *τ*_a,S_) significantly increased *A*_f_ to 24%. Therefore, small but significant differences in *τ* are sufficient to drive improvements in diurnal carbon gain.

**Table 2:** Impact of Rubisco deactivation and activation on potential diurnal net CO_2_ assimilation (*A*_diel_) of four *Vigna* accessions

Model	*Vigna* accession	*A*_diel_	*A*_f_	
		(mmol m^−2^ d^−1^)	(mmol m^−2^ d^−1^)	(%)
*A*_*Q*_	*V. adenantha*	523	–	–
	*V.* sp. Savi	531	–	–
	IT82E-16	569	–	–
	IT86D-1010	554	–	–
	Mean ± 95% CI	544 ± 18.3	–	–
*τ*_d,V_ *τ*_a,V_	*V. adenantha*	418	105	20.0
	*V.* sp. Savi	423	108	20.4
	IT82E-16	449	120	21.1
	IT86D-1010	465	113	20.4
	Mean ± 95% CI	433 ± 6.1	112 ± 7.9	20.5 ± 0.83
*τ*_d,S_ *τ*_a,S_	*V. adenantha*	422	101	19.4
	*V.* sp. Savi	419	113	21.2
	IT82E-16	455	114	20.1
	IT86D-1010	437	118	21.2
	Mean ± 95% CI	414 ± 6.1	111 ± 5	20.5 ± 0.91
*τ*_d,V_ *τ*_a,S_	*V. adenantha*	435	88	16.9
	*V.* sp. Savi	440	91	17.2
	IT82E-16	468	101	17.8
	IT86D-1010	459	95	17.1
	Mean ± 95% CI	450 ± 6.1	94 ± 5	17.2 ± 0.91
*τ*_d,S_ *τ*_a,V_	*V. adenantha*	405	118	22.6
	*V.* sp. Savi	401	131	24.6
	IT82E-16	435	134	23.6
	IT86D-1010	418	137	24.7
	Mean ± 95% CI	433 ± 6.1	130 ± 5	23.9 ± 0.91

Perfect tracking of changes in PPFD by net CO_2_ assimilation based on steady-state light-response curves (*A*_Q_), is compared with models in which the rate of change in Rubisco activity during deactivation (*τ*_d_) and activation (*τ*_a_) in response to fluctuating PPFD are alternatively parameterized using one-point *V*_c,max_ from leaf gas exchange (*τ*_-,V_) or Rubisco activation state (*S*; *τ*_-,S_). Foregone assimilation (*A*_f_) is the difference between *A*_Q_ and the respective alternative models.

New, high-frequency sampling during sun–shade transitions for biochemical analysis of Rubisco activation in cowpea, revealed far more rapid deactivation than previously appreciated on the basis of gas exchange measurements^2^. Modelling of these results augments predictions of 2–20% impacts of shade-induced changes in Rubisco activity on diurnal photosynthesis^2,3^. Prior estimates have relied on gas exchange in wheat^1^, where estimated *τ*_d_ was slightly longer than *τ*_a_, consistent with measurements using *S* in spinach, basil and impatiens^16,17^. Longer deactivation times, important for exploitation of sunflecks, have also been reported in the tropical understorey species *Alocasia macrorrhiza*^15^. By contrast, the fast decline in *S* measured in cowpea suggests that shade-induced Rubisco limitation may have been underestimated for some crops. An answer to the question of why cowpea does not exhibit longer deactivation times may be that its wild ancestors exploited warm, dry climates^29^ where shading was less important than in forest or contemporary cropping environments.

Using *S* to establish Rubisco activity in shade required a carefully constructed, laboratory-based set-up and more work is needed to understand the offset in *τ*_a_ and evidence that (de-)carbamylation rather than RuBP/inhibitor-binding drove Rubisco activity under our assay conditions. Gas exchange-based methods therefore remain the best option for evaluating Rubisco regulation in, for example, breeders plots^2,3,10,18,21,22^. Here, the use of equation (6) with a constrained point-estimate of *V*_c,max,L_ overestimated *τ*_d,V_. This will be improved by experiments that establish how *V*_c,max_ responds to shade periods of different durations. Our finding that *S* in cowpea stabilized within 10 min of shade also suggests use of <20 min of shade, with the benefit that stomatal closure would be less and so less complicating to gas exchange assays.

Significant variation in Rubisco deactivation half-times (*τ*_d,S_) among *Vigna* accessions suggests that *τ*_d_ would be amenable to selection for improvement in breeding programmes. Variation between two cowpea lines from the same geographical origin (IT86D-1010 and IT82E-16) also suggests that greater variation is probably available from more diverse germplasm. Induction is relatively easy to study using field portable gas exchange equipment, so has been a focus in recent studies highlighting Rubisco regulation in crop plants^2,3,10,21,22,27^;﻿ however, measurements of *S* suggest that, at least in cowpea, the speed of response to shade differs more than speed of induction. Slowing Rubisco deactivation during shade is a new target for crop improvement, with potential to improve productivity in food crops like cowpea.

## Methods

### Plant material and growth conditions

*V. unguiculata* (L.) Walp. IT86D-1010 and IT82E-16 (cowpea, obtained from the US Department of Agriculture), an interfertile wild relative *Vigna* sp. Savi TVNu-1948 (obtained from the International Institute of Tropical Agriculture; detailed information on IT and TVNu accessions can be obtained from https://my.iita.org/accession2/) and *V. adenantha* (G. Mey.) Marechal, Mascherpa & Stanier (wild pea, obtained from the Royal Botanic Gardens Millennium Seed Bank, Kew) were germinated in 0.6 l Deepots (D40H, Stuewe & Sons) containing a 1:1 (v:v) mixture of silver sand (horticultural grade, Royal Horticultural Society, London) and nutrient-rich compost (Petersfield Growing Mediums). Plants were grown for 3.5 weeks in a glasshouse, watered as needed to soil saturation and fertilized at 2 weeks (MiracleGro). Day/night temperatures were 28.2 ± 1.9 °C/19.2 ± 1.0 °C, with a photoperiod of 16 h and natural light supplemented to maintain a minimum PPFD of 500 µmol m^–2^ s^–1^.

### Sampling under changing irradiance for Rubisco activation

The leaf-disc method used is a variant of previously described light assays conducted in vitro^24,25^. The artificial sunlight simulation rig (light-rig; Supplementary Fig. 1) consisted of two high-intensity dimmable LED grow lights (Specialty Lighting Holland BV), jointly capable of supplying a PPFD of >1,200 µmol m^–2^ s^–1^ with a spectrum designed to closely match clear-sky solar irradiance. A steel frame allowed precise positioning of the lights and was enclosed on three sides using white reflective shielding to improve uniformity of lighting (MCPET M4, Furukawa Electric Europe). Light treatments were implemented using a SLESA-UE7 lighting controller incorporated into a custom control interface (Specialty Lighting Holland BV), programmed using the Easy Stand Alone 2 software (Nicolaudie Architectural Lighting). Leaf discs (0.55 cm^2^) were excised from intact plants in the glasshouse and immediately placed with the abaxial surfaces in contact with 25 mM MES-NaOH pH 5.5, in 50 ml beakers filled to within 5 mm of the rim, maintaining the usual orientation with respect to irradiance. A circulating water bath containing a 37 × 25 cm^2^ metal rack coated with non-reflective primer was used to hold the beakers in the light-rig. PPFD was measured at leaf-disc-level for each position within the rack to control uniformity of treatment levels and the water bath maintained the buffer at a constant temperature of 30 ± 0.1 °C (Omega Thermocouple Thermometer RDXL4SD, equipped with a type-K thermocouple; Omega). The rack had a 6 × 4 array to meet our randomized sampling design. Leaf discs were sampled for Rubisco assays by snap-freezing into liquid nitrogen after blotting on Whatman filter paper. Samples were stored at −80 °C until biochemical analysis. The sampling method by incubation of leaf discs at specific light and temperature conditions in the light-rig enabled accurate determination of Rubisco activity and activation state, representative of that found in intact leaves. Comparable results were obtained using leaf-disc samples collected from intact leaves and after incubation of leaf discs by floating in 25 mM MES-NaOH pH 5.5 or H_2_O for 60 min under the same light and temperature conditions in the glasshouse (Supplementary Fig. 3). The incubation time was also tested, with 20, 40 and 60 min producing comparable results (Supplementary Fig. 4). The source of CO_2_ to the leaf discs during incubation in the light-rig is the ambient air in contact with the adaxial leaf surface. Ambient air was circulated using two fans positioned at the top of the partially enclosed light-rig. In addition to the comparison with intact leaves, comparable Rubisco activation states in leaf discs floated in 25 mM MES-NaOH pH 5.5 with and without 10 mM NaHCO_3_ showed that leaf discs were not CO_2_ limited (Supplementary Fig. 5).

To establish the light response of Rubisco activation (Supplementary Fig. 2), one leaf disc per plant from four to six replicates of every genotype, was illuminated for 40 min at PPFD of 0, 80, 160, 240, 320, 400, 500, 850 and 1,200 µmol m^–2^ s^–1^. PPFD at the level of the leaf discs was measured before each assay (Q203 Quantum Radiometer with PFD filter, Irradian). Using the same system, time series were sampled to establish changes in Rubisco activation following sun–shade (deactivation) and shade–sun (activation) transitions. Each time series consisted of 32 discs collected from the youngest fully expanded trifoliate leaf on an individual plant. Treatments during time series consisted of high light for sun (850 µmol m^–2^ s^–1^ PPFD) for 40 min; low light for shade (150 µmol m^–2^ s^–1^ PPFD) for 20 min and a return to high light for sun (‘postshade’). Leaf discs were first sampled 1 and 3 min before the transition to shade. Then, during both the shade and postshade periods, discs were sampled every 15 s for 2 min, then every 2 min until 20 min after the change in irradiance.

### Rubisco activation state (*S*) measurements

Leaf samples (0.55 cm^2^) were ground in a mortar and pestle for up to 1 min in 250 µl of ice-cold buffer containing 50 mM Bicine-NaOH, pH 8.2, 20 mM MgCl_2_, 1 mM EDTA, 2 mM benzamidine, 5 mM ε-aminocaproic acid, 50 mM 2-mercaptoethanol, 10 mM DTT, 1 mM phenylmethylsulphonyl fluoride and 1% (v/v) protease inhibitors^30^. The leaf lysate was cleared by centrifugation (14,000*g* for 1 min) at 4 °C. The supernatant was collected into a new tube, quickly mixed by pipetting and immediately used to initiate the Rubisco reactions. Rubisco initial and total activities at 30 °C were measured by the incorporation of ^14^CO_2_ into 3-phosphoglycerate, following the carboxylation reaction by Rubisco^31^. Initial activities were obtained by adding 25 µl of supernatant to the assay mix containing 100 mM Bicine-NaOH, pH 8.2, 20 mM MgCl_2_, 10 mM [^14^C]-NaHCO_3_ (18.5 kBq µmol^–1^), 2 mM KH_2_PO_4_ and 0.6 mM RuBP. Total activities were obtained by incubating 25 µl of supernatant in the assay mix for 3 min, in the absence of RuBP. A test using IT68D-1010 showed the 3 min of incubation in the total activity assay was sufficient to allow available Rubisco catalytic sites to be carbamylated, resulting in the same *S* as 5 min of incubation (3 min, 79.7 ± 1.7%; 5 min, 79.6 ± 1.8%; *n* = 5). Reactions containing activated Rubisco were initiated by the addition of 0.6 mM RuBP. Both initial and total reactions were quenched after 30 s with 100 µl of 20% formic acid. Reaction vials were dried at 100 °C, rehydrated with 400 µl of ultrapure H_2_O, then mixed with 3.6 ml of scintillation cocktail (Gold Star Quanta, Meridian). Radioactive content of acid-stable ^14^C products was determined using a Liquid Scintillation Analyzer (Packard Tri-Carb, PerkinElmer). Rubisco activation state (*S*) is the ratio of initial to total Rubisco activity^32–34^.

### Leaf gas exchange

Photosynthesis in terminal leaflets of recently expanded first or second trifoliate leaves (Supplementary Fig. 6), consistent with material used for Rubisco activity assays, was characterized in the glasshouse using two portable gas exchange systems (LI-6800F Photosynthesis Systems LI-COR; with Bluestem v.1.2.2, Scripts v.2017.12 1.2.1, October 2017, and Fluorometer v.1.1.6); all genotypes being measured on each system. Steady-state gas exchange, assessed as a period of ≥5 min with no directional trend in the rate of leaf CO_2_ uptake was obtained with cuvette conditions of 1,500 μmol m^−2^ s^−1^ PPFD (40 μmol m^−2^ s^−1^ blue and 1,460 μmol m^−2^ s^−1^ red); 430 μmol mol^−1^ inlet [CO_2_]; leaf temperature 30.1 ± 0.45 °C (mean ± s.d., *n* = 105); and humidity controlled to achieve leaf vapour pressure deficit 1.48 ± 0.149 kPa. Combined gas exchange (CO_2_ and H_2_O) and chlorophyll fluorescence, using the multiphase flash protocol, measurements were made to establish the response of net CO_2_ assimilation (*A*) to [CO_2_] (430, 375, 300, 225, 150, 75, 30, recovery at 430, 500, 575, 625, 700, 800, 900, 1,000 μmol mol^−1^) and PPFD (1,500, 2,000, 1,700, 1,300, 1,100, 900, 700, 500, 400, 300, 200, 100, 50 and 0 μmol m^−2^ s^−1^). To establish the impact of shade on subsequent recovery of photosynthesis, gas exchange measurements were logged at 10 s intervals during steady-state; throughout a period of low light with equivalent light intensity (150 μmol m^−2^ s^−1^) and duration (20 min) to that used in Rubisco activity assays; and following return to the steady-state PPFD of 1,500 μmol m^−2^ s^−1^. Control of cuvette conditions during sun–shade–sun assays was achieved using set-points for air temperature (30 °C), relative humidity (60–70%, fixed at steady-state value) and CO_2_ supply (430 μmol mol^−1^).

### One-point estimates of Rubisco maximum carboxylation rates (*V*_c,max_)

The recovery of *V*_c,max_ following shade was predicted point-by-point from gas exchange measurements of *A* and *c*_i_ by rearranging the Farquhar et al.^35^ equation:

1 $$V_{{{{\mathrm{c}}}},{{{\mathrm{max}}}}} = \frac{{\left( {A + R_{{{\mathrm{d}}}}} \right)}}{{\left( {\frac{{c_{{{\mathrm{c}}}} - {\Gamma}^ \ast }}{{c_{{{\mathrm{c}}}} + K_{{{\mathrm{C}}}}\left( {1 + O/K_{{{\mathrm{O}}}}} \right)}}} \right)}}$$

where

2 $$c_{{{\mathrm{c}}}} = c_{{{\mathrm{i}}}} - \frac{A}{{g_{{{\mathrm{m}}}}}}$$

The parameters *R*_d_ (respiration in the light) and *g*_m_ (mesophyll conductance) were determined from steady-state *A*/*c*_i_ curves fit to the [CO_2_] assay data (measured from the same leaf and during the same diurnal period as induction measurements; Supplementary Fig. 7 and Supplementary Methods). For simplicity, *g*_m_ was assumed constant during induction, on the basis of recent measurements that show limited changes in *g*_m_ responding to similar sun–shade sequences that used 200 μmol m^−2^ s^−1^ as the shade irradiance in tobacco^36^. Parameters *K*_C_, *K*_O_ (Rubisco Michaelis–Menten coefficients for CO_2_ and O_2_, respectively) and Γ* (CO_2_ compensation point in the absence of *R*_d_) were predicted at the mean leaf temperature measured by the LI-6800F leaf thermocouple, using published equation sets for tobacco^37^. The concentration of O_2_ (*O*) was assumed to be the current atmospheric level of 209.5 mmol mol^−1^ and gas concentrations were converted to partial pressures before fitting the model.

### Statistical models of *S* and *V*_c,max_ time series

To obtain estimates of half-times for *S* and *V*_c,max_ in response to changes in light, the piecewise model of activation state was

3 $$S = a\left( {S_{{{\mathrm{H}}}}} \right) + b\left( {S_{{{\mathrm{L}}}} - \left( {S_{{{\mathrm{L}}}} - S_{{{\mathrm{H}}}}} \right)e^{\frac{{ - \left( {t + t_L} \right)}}{{\tau _{{{{\mathrm{d}}}},{{{\mathrm{S}}}}}}}}} \right) + c\left( {S_{{{\mathrm{H}}}} - \left( {S_{{{\mathrm{H}}}} - S_{{{\mathrm{L}}}}} \right)e^{\frac{{ - t}}{{\tau _{{{{\mathrm{a}}}},{{{\mathrm{S}}}}}}}}} \right)$$

where *a*, *b* and *c* are set to 1 in timesteps where the submodel is relevant: *a*, *t* ≤ −*t*_L_; *b*, −*t*_L_ < *t* ≤ 0; *c*, *t* > 0; and are otherwise set to 0. Time (*t*, s) is relative to the beginning of induction and *t*_L_ is the duration of low light (shade). Transitions between the steady-state Rubisco activity in high light (*S*_H_) and low light (*S*_L_) follow exponential trajectories. The coefficient determining the rate of decline in *S* after a high- to low-light transition (deactivation) is the half-time *τ*_d,S_; conversely, the half-time *τ*_a,S_ determines the rate of increase in *S* following transition from low to high light (activation).

The response of *V*_c,max_ reflecting Rubisco activation during induction was modelled as

4 $$V_{{{{\mathrm{c}}}},{{{\mathrm{max}}}}} = V_{{{{\mathrm{c}}}},{{{\mathrm{max}}}},{{{\mathrm{H}}}}} - \left( {V_{{{{\mathrm{c}}}},{{{\mathrm{max}}}},{{{\mathrm{H}}}}} - V_{{{{\mathrm{c}}}},{{{\mathrm{max}}}},{{{\mathrm{L}}}}}} \right)e^{\frac{{ - t}}{{\tau _{{{{\mathrm{a}}}},{{{\mathrm{V}}}}}}}}$$

where *V*_c,max,H_ and *V*_c,max,L_ are high-light and low-light steady-state values, respectively; and *t* is time from the start of shade (s). The rate of increase in *V*_c,max_ declines exponentially with half-time *τ*_a,V_. This model was fit to data collected between 1 and 5 min after shade, a period that followed the initial inflection in *A* associated with the end of the RuBP-regeneration phase and during which photosynthesis was determined to be consistently limited by *V*_c,max_ (Supplementary Fig. 8).

To establish the half-time for decrease in *V*_c,max_ on transfer to shade (*τ*_d,V_), we used the equation for decreasing *V*_c,max_

5 $$V_{{{{\mathrm{c}}}},{{{\mathrm{max}}}}} = V_{{{{\mathrm{c}}}},{{{\mathrm{max}}}},{{{\mathrm{L}}}}} - \left( {V_{{{{\mathrm{c}}}},{{{\mathrm{max}}}},{{{\mathrm{L}}}}} - V_{{{{\mathrm{c}}}},{{{\mathrm{max}}}},{{{\mathrm{H}}}}}} \right)e^{\frac{{ - t_{{{\mathrm{L}}}}}}{{\tau _{{{{\mathrm{d}}}},{{{\mathrm{V}}}}}}}}$$

Rearranging and taking logs provides an expression for *τ*_d,V_,

6 $$\tau_{{\mathrm{d}},{\mathrm{V}}} = \frac{-t_{\mathrm{L}}}{{\mathrm{ln}}\left(\left(V_{{\mathrm{c}},{\mathrm{max}},{\mathrm{L}}}-V_{{\mathrm{c}},{\mathrm{max}}} \right)/\left(V_{{\mathrm{c}},{\mathrm{max}},{\mathrm{L}}}-V_{{\mathrm{c}},{\mathrm{max}},{\mathrm{H}}} \right)\right)}$$

Further assuming that *V*_c,max_ at the end of 20 min shade is ~*V*_c,max,L_ + 1 (for context, 95% CI of *V*_c,max,L_ are ±17–22 μmol m^−2^ s^−1^; Table 1), so that *V*_c,max,L_ − *V*_c,max_ = −1, simplifies to

7 $$\tau _{{{{\mathrm{d}}}},{{{\mathrm{V}}}}} = \frac{{t_{{{\mathrm{L}}}}}}{{\mathrm{ln}\left( {V_{{{{\mathrm{c}}}},{{{\mathrm{max}}}},{{{\mathrm{H}}}}} - V_{{{{\mathrm{c}}}},{{{\mathrm{max}}}},{{{\mathrm{L}}}}}} \right)}}$$

This is an upper limit on *τ*_d,V_, the value of which decreases as *V*_c,max,L_ − *V*_c,max_ → 0.

Nonlinear-least-squares models were initially fit to individual replicates, providing starting models (*S*, Supplementary Table 2; *V*_c,max_, Supplementary Table 3) from which we aimed to identify significant differences at the level of accessions, the level relevant to crop improvement. Differences between the starting models were used to inform construction and simplification of nonlinear mixed-effects models (*S*, Supplementary Fig. 9; *V*_c,max_, Supplementary Fig. 10). Maximal models, that is complete parameterization at the level of individual replicates, with individuals treated as random effects, were progressively simplified. Using evidence from likelihood ratio testing, Wald tests and plots of residuals and model coefficients, fixed effects were introduced, their importance established and unnecessary fixed or random terms removed^38^.

### Diurnal assimilation models

The diurnal impact of shade-responsive changes in Rubisco activity on potential *A*, was predicted on the basis of fitted net CO_2_ assimilation-light-responses (A/PPFD) (Table 1, Supplementary Fig. 11 and Supplementary Methods) and an irradiance regime relevant to chloroplasts in second-layer leaves of a legume crop (Fig. 2a): irradiance values had been derived at ~60 s intervals by reverse ray tracing, with shade-generating structures in the canopy distributed at random within each layer and assuming a clear-sky day in June at latitude 44° N (ref. ^16^).

When PPFD was increasing, Rubisco limited *A* (*A*_R_) was modelled as^16^

8 $$A_{{{\mathrm{R}}}} = A_{{{\mathrm{F}}}} - \left( {A_{{{\mathrm{F}}}} - A_{{{\mathrm{I}}}}} \right)e^{\frac{{ - t}}{{\tau _{{{\mathrm{a}}}}}}}$$

The rate of change in *A*_R_ decreases exponentially over the duration of each timestep (*t*) in proportion to the Rubisco activation half-time (*τ*_a_). The net CO_2_ assimilation rate at the final PPFD (*A*_F_) is approximated using the PPFD response

9 $$A_{{{\mathrm{F}}}} = \frac{{\phi Q + A_{{{{\mathrm{sat}}}}} - \sqrt {\left( {\phi Q + A_{{{{\mathrm{sat}}}}}} \right)^2 - 4\phi {\uptheta}QA_{{{{\mathrm{sat}}}}}} }}{{2{\uptheta}}} - R_{{{\mathrm{d}}}}$$

where *ϕ* is an initial slope, *Q* is PPFD, *A*_sat_ is the light-saturated rate and *θ* a curvature parameter. In each timestep, the initial net CO_2_ assimilation rate (*A*_I_) is the *A*_R_ achieved at the end of the previous timestep (taken to be 0 at first light).

Assuming that [RuBP] is saturating, integrated, Rubisco activity-limited CO_2_ assimilation (${\int}_0^t A$, annotated as *A*_*τ*_) is

10 $$A_\tau = A_{{{\mathrm{F}}}}t - \left( {A_{{{\mathrm{F}}}} - A_{{{\mathrm{I}}}}} \right)\tau _{{{\mathrm{a}}}} + \left( {A_{{{\mathrm{F}}}} - A_{{{\mathrm{I}}}}} \right)\tau _{{{\mathrm{a}}}}e^{\frac{{ - t}}{{\tau _{{{\mathrm{a}}}}}}}$$

Setting *τ*_a_ = 0 integrates potential assimilation rate with instantaneous response to PPFD/quantum input (*A*_Q_ = *A*_F_*t*). An estimate of foregone assimilation, *A*_f_, is *A*_Q_ − *A*_τ_ (refs. ^16,26^), which is expressed as a percentage of potential assimilation:

11 $$A_{{{\mathrm{f}}}} = \frac{{A_{{{\mathrm{Q}}}} - A_\tau }}{{A_{{{\mathrm{Q}}}}}}100$$

When PPFD was decreasing, CO_2_ assimilation was modelled as responding immediately to PPFD: *A*_τ_ = *A*_Q_. However, to provide an appropriate *A*_I_ on return to non-light-limiting conditions, we predicted *A*_R_ as declining at a rate determined by *τ*_d_:

12 $$A_{{{\mathrm{R}}}} = A_{{{\mathrm{I}}}} - \left( {A_{{{\mathrm{I}}}} - A_{{{\mathrm{F}}}}} \right)e^{\frac{{ - t}}{{\tau _{{{\mathrm{d}}}}}}}$$

Outcomes of diurnal modelling (*A*_Q_ and *A*_f_) were compared using linear mixed effects, treating models using alternative (estimated from *S* or *V*_c,max_) *τ*_a_ and *τ*_d_ as fixed effects, while accounting for variation among accessions as a random effect.

### Statistical software

Modelling and statistical analyses were implemented in R (v.4.0.3; ref. ^39^) including the nlme package (v.3.1-151; ref. ^40^).

### Reporting Summary

Further information on research design is available in the Nature Research Reporting Summary linked to this article.

## Supplementary information

Supplementary Information Supplementary Methods, Tables 1–3, Figs. 1–11 and references.

Reporting Summary

## Data Availability

Data, including those shown in Figs. 1 and 2 and Supplementary Information are available through the Lancaster University data repository (10.17635/lancaster/researchdata/493)^41^.

## Code availability

Code used for analysis and figure preparation are available through GitHub (https://github.com/smuel-tylor/Fast-Deactivation-of-Rubisco); data can also be obtained from this location.
