# Author Correction: Faster than expected Rubisco deactivation in shade reduces cowpea photosynthetic potential in variable light conditions

**DOI:** 10.1038/s41477-022-01243-6

**Published:** 2022-08-22

**Authors:** Samuel H. Taylor, Emmanuel Gonzalez-Escobar, Rhiannon Page, Martin A. J. Parry, Stephen P. Long, Elizabete Carmo-Silva

**Affiliations:** 1 Lancaster Environment Centre, Lancaster University, Lancaster, UK; 2 Departments of Plant Biology and of Crop Sciences, Carl R. Woese Institute of Genomic Biology, University of Illinois, Urbana, IL USA

**Keywords:** Plant physiology, C3 photosynthesis, Rubisco, Natural variation in plants, Light responses

Correction to: *Nature Plants* 10.1038/s41477-021-01068-9, published online 20 January 2022.

During the final revision of the manuscript for publication we added an intermediate step to the derivation of equation (7). Unfortunately, the derivation as described is mathematically incorrect. We have corrected this error which results in no change to the calculations or results. There is, however, a subtle shift in interpretation of the derived parameter, τ_d,V_. Previously, we treated τ_d,V_ as an upper limit that was ‘likely’ to be an overestimate. We now understand these upper limits to be explicit overestimates that are nonetheless constrained to be within a reasonable range. Text changes were made in paragraphs 8, 10 and 12, and in the derivation of equation (7) from equation (5).

