# Supplementary material for: Faster than expected Rubisco deactivation in shade reduces cowpea photosynthetic potential in variable light conditions
Source: Nat Plants. 2022 Jan 20;8(2):118–24. doi: 10.1038/s41477-021-01068-9 (PMC8863576; doi:10.1038/s41477-021-01068-9)
Supplement: Supplementary file 1 — Supplementary Methods, Tables 1–3, Figs. 1–11 and references. [file 41477_2021_1068_MOESM1_ESM.pdf]

nature portfolio

https://doi.org/10.1038/s41477-021-01068-9

Supplementary information

Faster than expected Rubisco deactivation in shade reduces cowpea photosynthetic potential in variable light conditions

In the format provided by the authors and unedited

Supplementary Materials for:

Faster than expected Rubisco deactivation in shade reduces cowpea photosynthetic potential in variable light conditions

Samuel H Taylor[1,*], Emmanuel Gonzalez-Escobar[1,*], Rhiannon Page[1], Martin AJ Parry[1], Stephen P Long[1,2], Elizabete Carmo-Silva[1]

[1] Lancaster Environment Centre, Lancaster University, Lancaster, LA1 4YQ, UK

[2] Departments of Plant Biology and of Crop Sciences, Carl R. Woese Institute of Genomic Biology, University of Illinois, 1206 W. Gregory Dr., Urbana, IL 61801, USA

* Authors contributed equally to this work.

Corresponding author. E-mail: e.carmosilva@lancaster.ac.uk

Supplementary Methods 2
- A/PPFD responses 2
- A/c_i responses 2

Supplementary Tables 4
- Table S1. List of abbreviations 4
- Table S2. Coefficients from Individual-by-individual models used as a starting point for mixed effects modelling of time-series for *S* 5
- Table S3. Coefficients from Individual-by-individual models used as a starting point for mixed effects modelling of time-series for $V_{c,max}$ 5

Supplementary Figures 6
- Fig. S1. Schematic of the lighting system 6
- Fig. S2. Light responses for Rubisco activation state 7
- Fig. S3. Comparison of leaf discs obtained from intact plants and assayed *in vitro* under greenhouse conditions. 8
- Fig. S4. Comparing methods for assaying Rubisco activities from cowpea leaf discs. 9
- Fig. S5. The effect of 10 mM $NaHCO_3$ on Rubisco activation state in *Vigna* accession leaf discs incubated in 25 mM MES using the phenotyping light rig. 10
- Fig. S6. Plant status and leaf choice at time of sampling 11
- Fig. S7. Steady state A/c_i responses 12
- Fig. S8. Duration of Rubisco limitation 16
- Fig. S9. Sun-shade-sun responses for Rubisco activation state (*S*) 20
- Fig. S10. Induction (shade-sun) responses for one-point $V_{c,max}$ 24
- Fig. S11. A/PPFD responses 28

References 32

Supplementary Methods

A/PPFD responses

The relationship between A and incident PPFD was modelled as a non-rectangular hyperbola[1,2]:

$$A = \frac{\phi I + A_{\mathrm{sat}} - \sqrt{(\phi I + A_{\mathrm{sat}})^2 - 4\theta\phi I A_{\mathrm{sat}}}}{2\theta} - R_{\mathrm{d}}$$

Where: φ is the apparent quantum yield (mol mol^{-1}); *I*, incident PPFD (µmol m^{-2} s^{-1}); A_{sat}, the maximum gross rate of leaf CO_2 assimilation (µmol m^{-2} s^{-1}); θ, a dimensionless curvature parameter; and R_{d}, day respiration (µmol m^{-2} s^{-1}). Parameters were obtained using the optimiser function *optim* in R Language and Environment (v3.6.3[3]).

A/c_{i} responses

The FvCB model[4,5] was used to characterise *A*/c_{i} relationships:

$$A = min\left(W_{\mathrm{C}}, W_{\mathrm{J}}, W_{\mathrm{P}}\right)(1 - \Gamma^*/c_{\mathrm{c}}) - R_{\mathrm{d}}$$

$$W_{\mathrm{C}} = V_{\mathrm{c,max}} c_{\mathrm{c}}/(c_{\mathrm{c}} + K_{\mathrm{CO}})$$

$$W_{\mathrm{J}} = J c_{\mathrm{c}}/(4c_{\mathrm{c}} + 8\Gamma^*)$$

$$W_{\mathrm{P}} = 3T_{\mathrm{P}}\, c_{\mathrm{c}}/(c_{\mathrm{c}} - \Gamma^*)$$

where W_{C} is the Rubisco limited, W_{J} electron transport limited, and W_{P} triose-phosphate utilisation limited rate of carboxylation. The [CO_2] at the site of carboxylation in the chloroplast, $c_c = c_i - A/g_m$. Additional parameters are: Γ^*, the photosynthetic CO_2 compensation point in the absence of R_{d}; $V_{\mathrm{c,max}}$, the maximum carboxylation rate of Rubisco; $K_{\mathrm{CO}} = K_{\mathrm{C}}(1+O/K_{\mathrm{O}})$, where K_{C} and K_{O} are the respective Michaelis constants for Rubisco catalysis of carboxylation and oxygenation reactions, and *O* is the partial pressure of O_2; *J*, electron transport rate; T_{P}, the rate of triose phosphate utilisation.

With values for [CO_2] in partial pressure units, the match between c_{i} and W_{C}, W_{J}, and W_{P} as limiting factors was identified using a previously published approach[6,7]. Values for $V_{\mathrm{c,max}}$, *J*, and T_{P} were fit using:

$$A = \frac{\mathrm{b} - \sqrt{b^2 - 4c}}{2}$$

For A_{C}:

$$b = V_{c,max} - R_d + g_m(c_i + K_{CO})$$

$$c = g_m\left(V_{c,max}(c_i - \Gamma^*) - R_d(c_i + K_{CO})\right)$$

For A_{J}:

$$b = J/4 - R_d + g_m(c_i + 2\Gamma^*)$$
$$c = g_m\big(J/4(c_i - \Gamma^*) - R_d(c_i + 2\Gamma^*)\big)$$

For A_P:

$$b = 3T_p - R_d + g_m(c_i - \Gamma^*)$$
$$c = g_m\big(3T_P(c_i - \Gamma^*) - R_d(c_i - \Gamma^*)\big)$$

For each A/c_i response, all possible limitation-state combinations were tested, respecting the required order of limitation states along the c_i axis ($W_C < W_J < W_P$) and the minimum number of data necessary for each limitation state ($n \geq 2$ when K_{CO} and Γ^* are fixed). The distribution-wise cost function was minimised using *optim*[3], accepting the model with the lowest value after checking for admissibility and testing for co-limited 'swinging points'[6].

Preliminary fitting of different sets of parameters in the model showed that reasonable, positive, and non-zero estimates of R_d could be obtained by fixing g_m to 5 µmol m^{-2} s^{-1} Pa^{-1} and using published temperature dependencies of Γ^* K_C, and K_O from tobacco[8]. The fit of limitation state assignments was validated qualitatively using the increase (W_C), saturation (W_J) and decrease (W_P) in Φ_{PSII} with c_i[9]. The effective quantum yield, $\Phi_{PSII} = (F_m' - F_s)/F_m'$, having been derived from background (F_s) and maximum (F_m') fluorescence yields obtained with a multiphase flash using the integrated fluorometer in the LI-6800F cuvette.

Supplementary Tables

Table S1. List of abbreviations

3-PGA	3-phosphoglycerate
A	net CO_2 assimilation rate
a	binary coefficient for piecewise model of sun-shade-sun response by Rubisco activation state
A_f	foregone assimilation (diurnal)
A_F	net CO_2 assimilation rate at a final PPFD (for a timestep in diurnal model)
A_I	net CO_2 assimilation rate at an initial PPFD (for a timestep in diurnal model)
A_Q	integrated net CO_2 assimilation (for a timestep in diurnal model) as an instantaneous response to PPFD, i.e. ignoring τ
A_R	Rubisco limited net CO_2 assimilation rate
A_{sat}	light saturated net CO_2 assimilation rate in the *A*/PPFD relationship modelled as a non-rectangular hyperbola
A_τ	integrated net CO_2 assimilation (for a timestep in diurnal model), accounting for τ
b	binary coefficient for piecewise model of sun-shade-sun response by Rubisco activation state
c	binary coefficient for piecewise model of sun-shade-sun response by Rubisco activation state
g_m	mesophyll conductance (to CO_2)
I	irradiance, as PPFD, in the A/PPFD relationship modelled as a non-rectangular hyperbola
K_C	Michaelis-Menten coefficient for Rubisco carboxylation
K_O	Michaelis-Menten coefficient for Rubisco oxygenation
O	atmospheric concentration of O_2
PMSF	phenylmethylsulphonyl fluoride
PPFD	photosynthetic photon flux density
Rca	Rubisco activase
R_d	respiration in the light
RuBP	ribulose-1,5-bisphosphate
S	Rubisco activation state, V_i/V_t
S_H	steady-state Rubisco activation state at high light (sun)
S_L	steady-state Rubisco activation state at low light (shade)
t	time
t_L	the duration of low light (shade) affecting Rubisco activation state
V_i	Rubisco initial activity measured *in vitro*
V_t	Rubisco total activity measured *in vitro*
$V_{c,max}$	gas exchange estimate of the rate of carboxylation by Rubisco
$V_{c,max,H}$	asymptotic value of maximum Rubisco carboxylation rate in high light (sun) following induction
$V_{c,max,L}$	maximum Rubisco carboxylation rate after 20 min low light (shade) prior to induction
Γ^*	CO_2 compensation point in the absence of R_d
θ	non-dimensional curvature parameter in the *A*/PPFD relationship modelled as a non-rectangular hyperbola
τ	a half time
τ_a	half-time for activation of Rubisco
$\tau_{a,S}$	half-time for activation of Rubisco based on Rubisco activation state (*S*)
$\tau_{a,V}$	half-time for activation of Rubisco based on gas exchange estimates of maximum Rubisco carboxylation rate ($V_{c,max}$)
τ_d	half-time for deactivation of Rubisco
$\tau_{d,S}$	half-time for deactivation of Rubisco based on Rubisco activation state (*S*)
$\tau_{d,V}$	half-time for deactivation of Rubisco based on gas exchange estimates of maximum Rubisco carboxylation rate ($V_{c,max}$)
φ	initial slope of the *A*/PPFD relationship modelled as a non-rectangular hyperbola

Table S2. Coefficients from Individual-by-individual models used as a starting point for mixed effects modelling of time-series for *S*

Sample	S_L (%)	S_H (%)	$\tau_{d,S}$ (s)	$\tau_{a,S}$ (s)
*V. adenantha*_A	61.17806	79.05034	20.000072	207.82914
*V. adenantha*_C	60.09176	81.64326	181.421458	100.53259
*V. adenantha*_D	58.97032	85.42016	229.449731	455.91706
V. sp. Savi_A	54.82047	63.65896	6.455822	39.59381
V. sp. Savi_B	54.36652	61.62611	8.915565	137.31111
V. sp. Savi_C	55.20694	69.95726	52.927607	216.83621
V. sp. Savi_D	47.78410	67.81300	61.956361	166.24391
IT82E-16_B	57.14931	69.37678	265.740942	107.12442
IT82E-16_C	49.60266	73.21978	102.549414	145.01491
IT82E-16_D	53.20158	69.65418	160.822643	161.09552
IT86D-1010_A	59.68661	79.93667	65.382802	113.33060
IT86D-1010_B	57.60660	80.13056	23.677550	137.39551
IT86D-1010_C	61.16164	79.58986	19.390893	142.53163
IT86D-1010_D	53.79355	80.79573	70.550586	106.86711
*Letter coding alongside genotype names indicates experimental blocks (not used in analysis)				

Table S3. Coefficients from Individual-by-individual models used as a starting point for mixed effects modelling of time-series for $V_{c,max}$

Sample*	$V_{c,max,L}$ (%)	$V_{c,max,H}$ (%)	$\tau_{a,V}$ (s)
*V. adenantha*_2	42.54193	151.4440	95.13726
*V. adenantha*_5	110.96318	273.3067	185.23528
*V. adenantha*_6	130.85351	282.7727	350.97213
*V. adenantha*_9	97.49639	278.2085	184.21776
*V. sp. Savi*_1	78.96409	197.2805	113.80807
*V. sp. Savi*_2	77.20333	172.1294	174.78790
*V. sp. Savi*_5	70.40896	316.6256	91.00129
*V. sp. Savi*_6	117.72291	210.1258	183.68795
*V. sp. Savi*_8	23.57169	234.5373	98.92535
*V. sp. Savi*_9	126.77560	294.3917	213.21886
IT82E-16_1	102.86741	219.1356	161.29920
IT82E-16_2	142.55706	309.3509	824.59265
IT82E-16_5	141.38060	260.1907	201.56364
IT82E-16_6	146.07970	299.8841	155.52270
IT82E-16_8	61.07451	176.2086	104.30483
IT82E-16_9	88.36712	221.0774	100.91744
IT86D-1010_1	93.01146	243.2872	147.46197
IT86D-1010_6	167.34697	306.9400	390.30264
IT86D-1010_8	107.40724	257.4983	215.76208
IT86D-1010_5	106.87984	179.2362	150.68602
* Number coding alongside genotype names indicates experimental blocks (not used in analysis)			

Supplementary Figures

Fig. S1. Schematic of the lighting system

Artificial sunlight simulation rig schematic (A) and in operation (B) for phenotyping diversity in Rubisco activity and activation using a modified leaf disc method approach. Two lab-jacks allow re-positioning of the water bath to achieve varying light intensities. Freshly excised leaf discs in 50 mL beakers containing 25 mM MES pH 5.5 are exposed to specific PPFD and temperature conditions prior to sampling. Easy access to liquid nitrogen dewar allows rapid snap-freezing of leaf discs after collection from assay beakers.

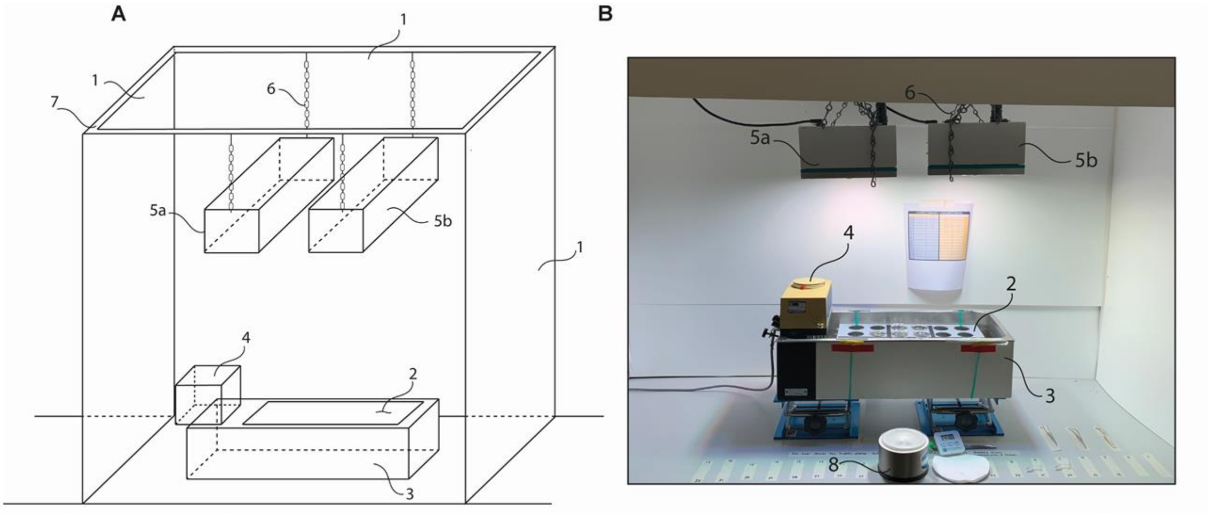

1, reflective shielding; **2,** rack to hold 50 mL beakers; **3,** water bath; **4,** temperature controlled water bath heater; **5,** LED growth lights; **6,** chains to hold the LED lights; **7,** metal structural support beam; **8,** liquid nitrogen dewar for snap-freezing leaf discs.

Fig. S2. Light responses for Rubisco activation state

Light response curves from *Vigna* accessions following changes in Rubisco activation at 30 °C (main document Methods) with increasing light intensity, using the light-rig. Cowpea (*V. unguiculata)* accessions (a) IT86D-1010 and (b) IT82E-16; cowpea wild relatives (c) *V. sp. Savi*. (TVNu-1948) and (d) wild pea *V. adenantha*. Shading indicates the standard error of the mean around the loess regression trendline.

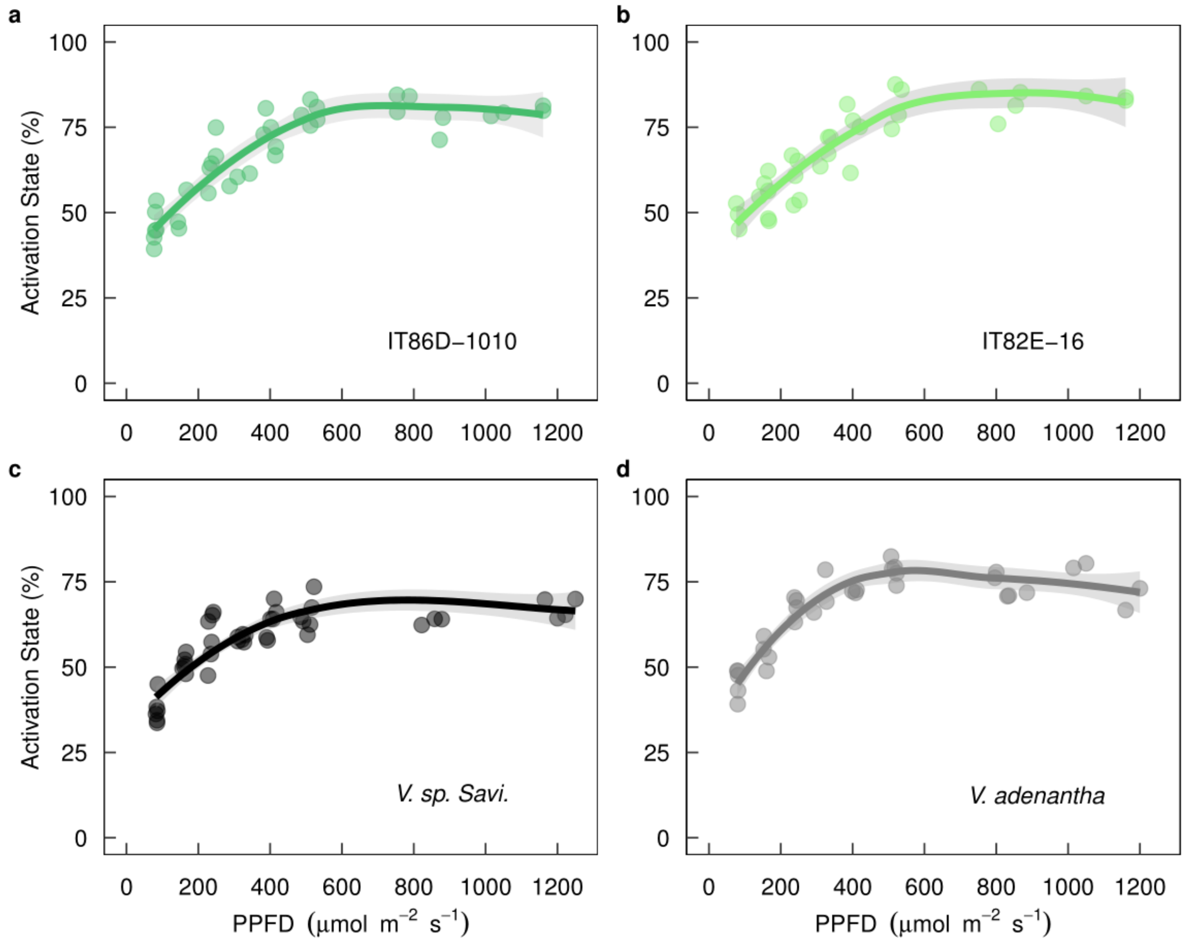

Fig. S3. Comparison of leaf discs obtained from intact plants and assayed *in vitro* under greenhouse conditions.

Rubisco activation state (a), initial (b) and total (c) activities (main document Methods) were measured at 30 °C from leaf discs of cowpea accession IT86D-1010. These were either directly frozen from 3.5-weeks old intact plants grown at 400 μmol m^{-2} s^{-1}, or frozen after 60 min flotation at the same light intensity in greenhouse conditions, using either a solution of 25 mM MES-NaOH, pH 5.5, or using distilled water, pH 7 for 60 min. Replicates were excised from terminal and lateral leaflets of the 2nd fully expanded trifoliate leaf of four individual plants. Material used in different test conditions originated from the same plant. There were no significant differences between test conditions (bold letters, one-way ANOVA, P > 0.05). Boxplots indicate medians, 25th & 75th percentile and, where relevant, whiskers indicate values falling within 1.5 × the interquartile range. All Individual data are also plotted as points.

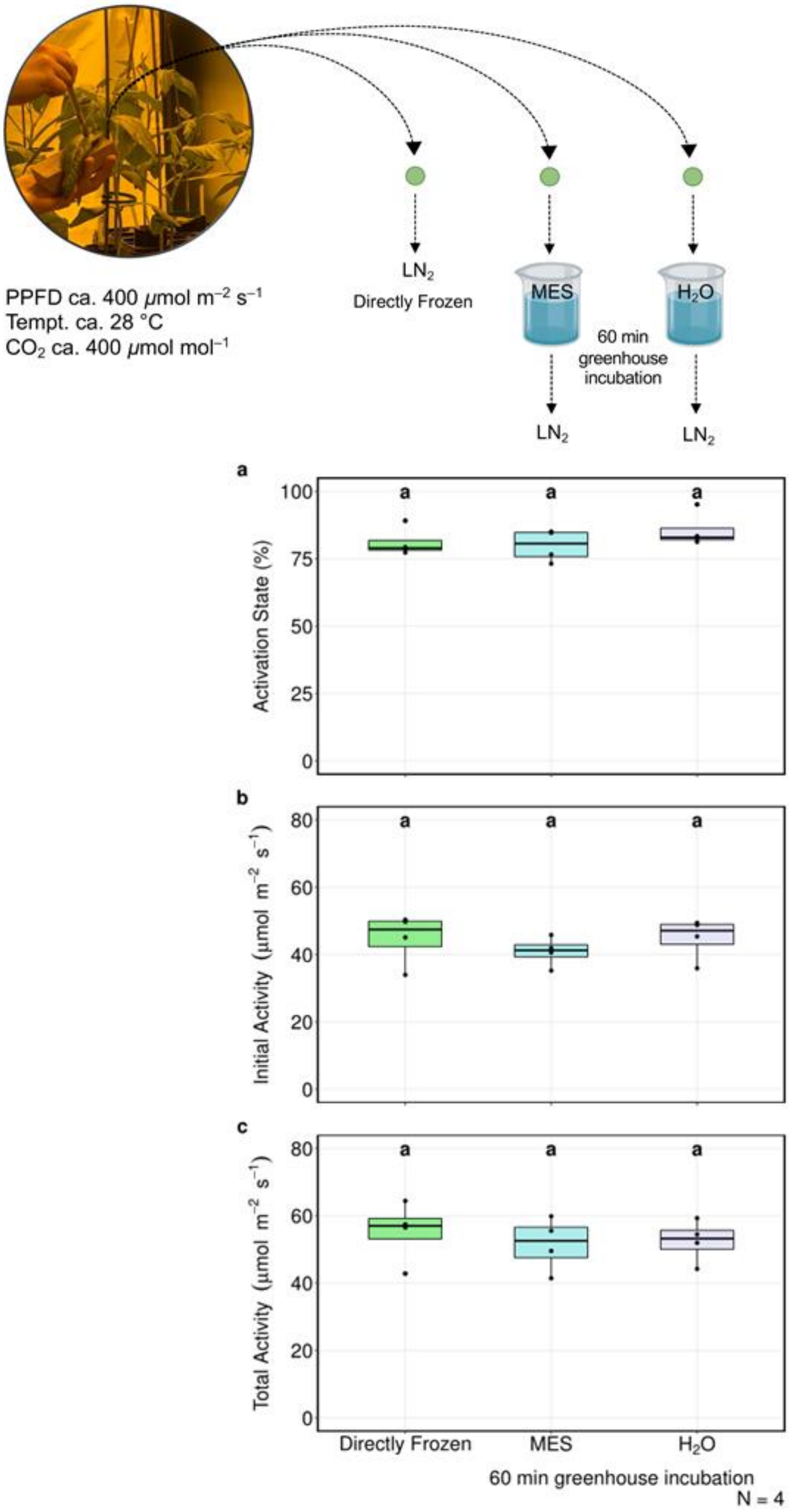

Fig. S4. Comparing methods for assaying Rubisco activities from cowpea leaf discs. Rubisco activation state (a), initial (b) and total (c) activities (main document Methods) were determined at 30 °C. Leaf discs samples from cowpea accession IT86D-1010 were incubated in 25 mM MES buffer in the light-rig for 20, 40 and 60 min at 500 μmol m^{-2} s^{-1}. Letters indicate the results of a one-way ANOVA: Rubisco Activation State was lower with only 20 min incubation (40 min P = 0.07; 60 min P = 0.018), but there were no significant differences in Activity across the three incubation times. Boxplot statistics as Fig. S3.

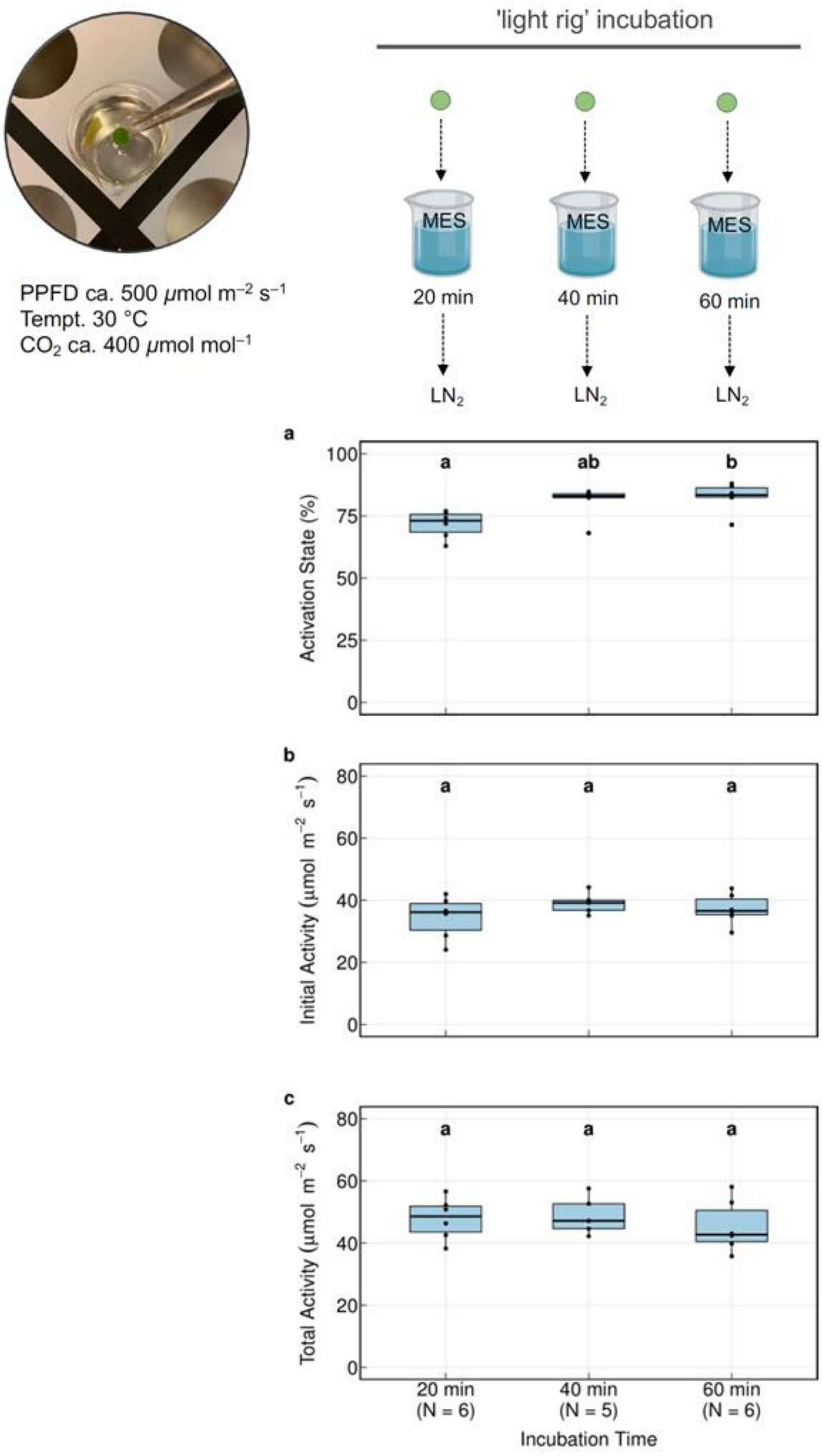

Fig. S5. The effect of 10 mM $NaHCO_3$ on Rubisco activation state in *Vigna* accession leaf discs incubated in 25 mM MES using the phenotyping light rig.

Rubisco activation states at 30 °C (main document Methods) from two cowpea (IT86D-1010 and IT82E-16) and wild pea accessions (*V. adenantha, V. sp. Savi*) were obtained after incubation with and without the addition of 10 mM $NaHCO_3$, after 40 and 80 min incubation at 1000 µmol m^{-2} s^{-1} using the light-rig. A repeated measures three-way ANOVA comparing incubation time and $NaHCO_3$ concentration by accession, found no significant differences between buffer types after 80 min incubation, irrespective of accession. The same was true when using 40 min incubation time, for all accessions but *V. adenantha*, leaf discs of which showed significantly lower activation state in MES than with addition of $NaHCO_3$. Boxplot statistics as Fig. S3.

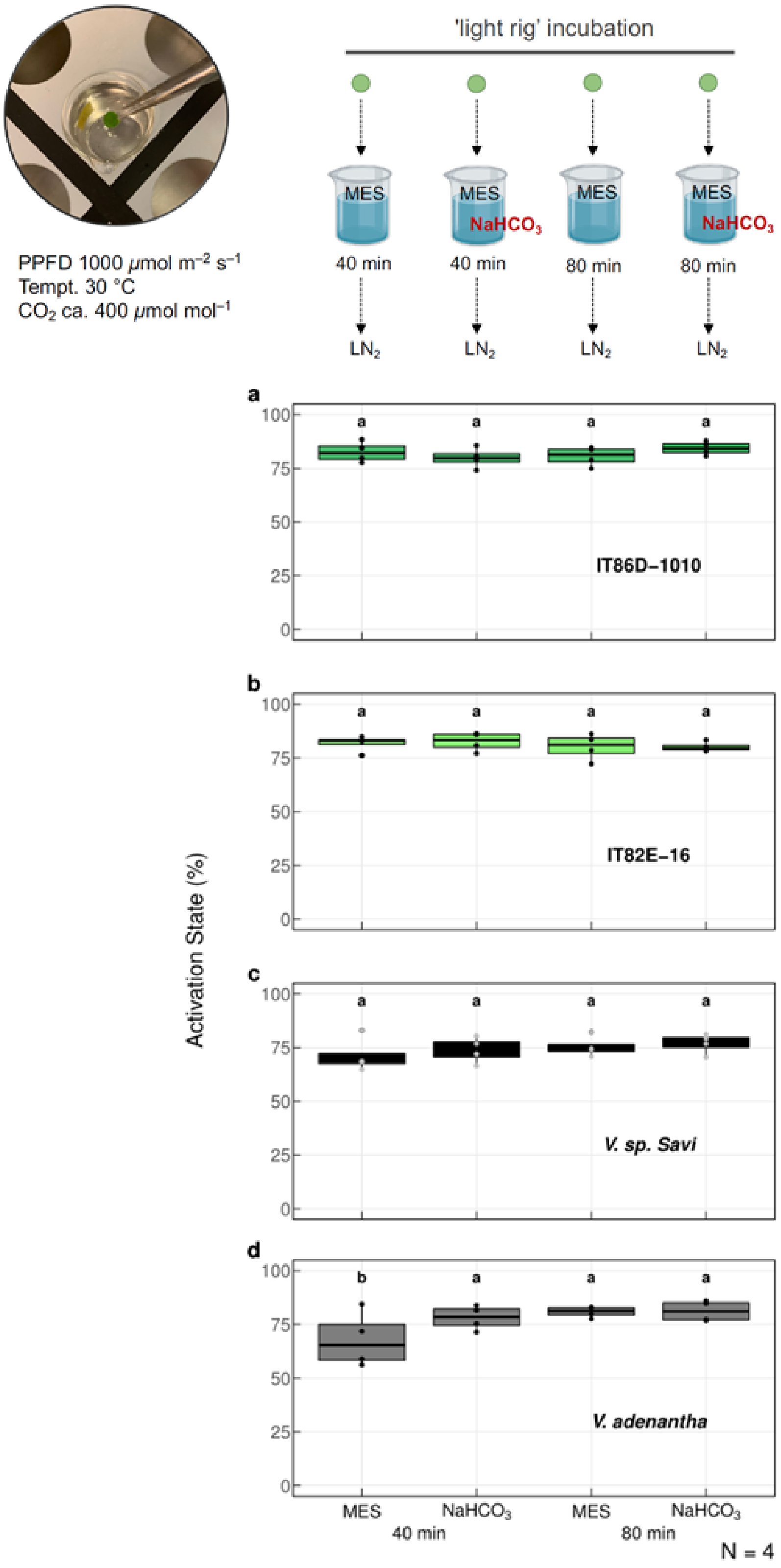

Fig. S6. Plant status and leaf choice at time of sampling
Measurements were carried out on fully expanded leaves 3-4 weeks after planting, at which stage the youngest fully expanded leaves were the first or second true leaves. Leaf expansion was judged based on colour and texture, with leaves judged to be fully expanded once they were dark green and glossy, an appearance that matched that of older leaves but not younger, expanding foliage.

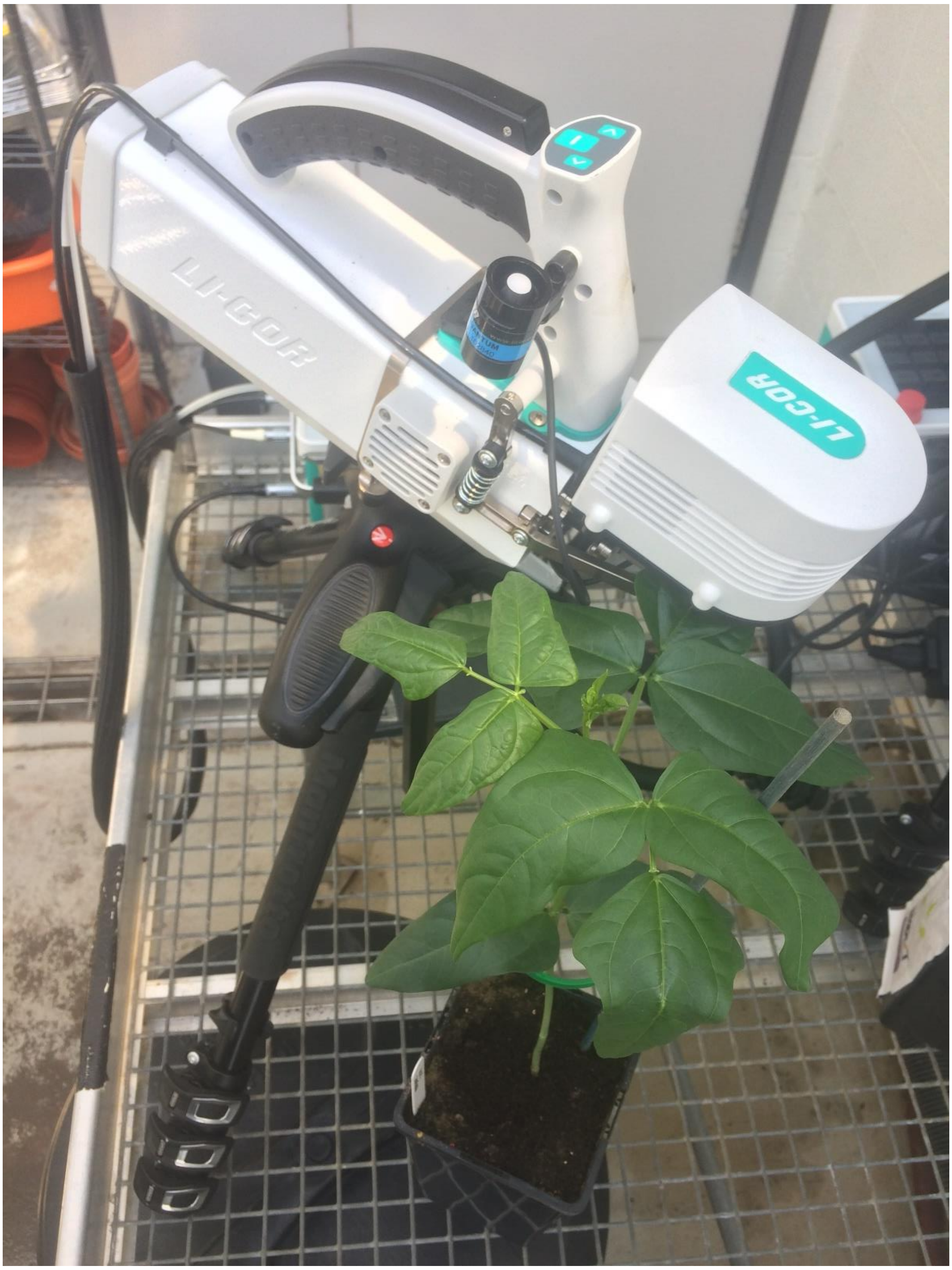

Fig. S7. Steady state A/c_i responses

See Supplementary Methods for details. Overplotted red symbols indicate the steady state operating point. Individuals coded as in Table S3.

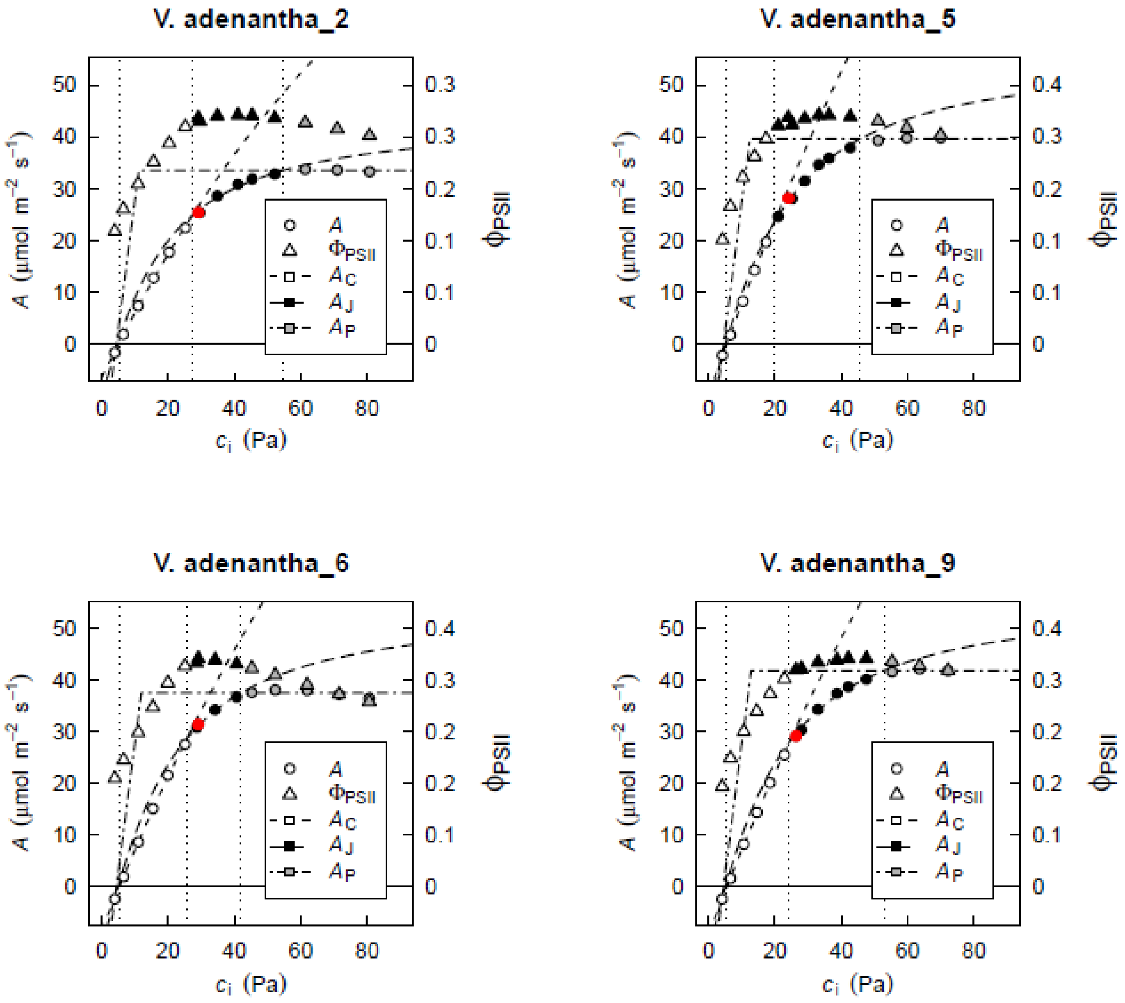

Fig. S7 continued.

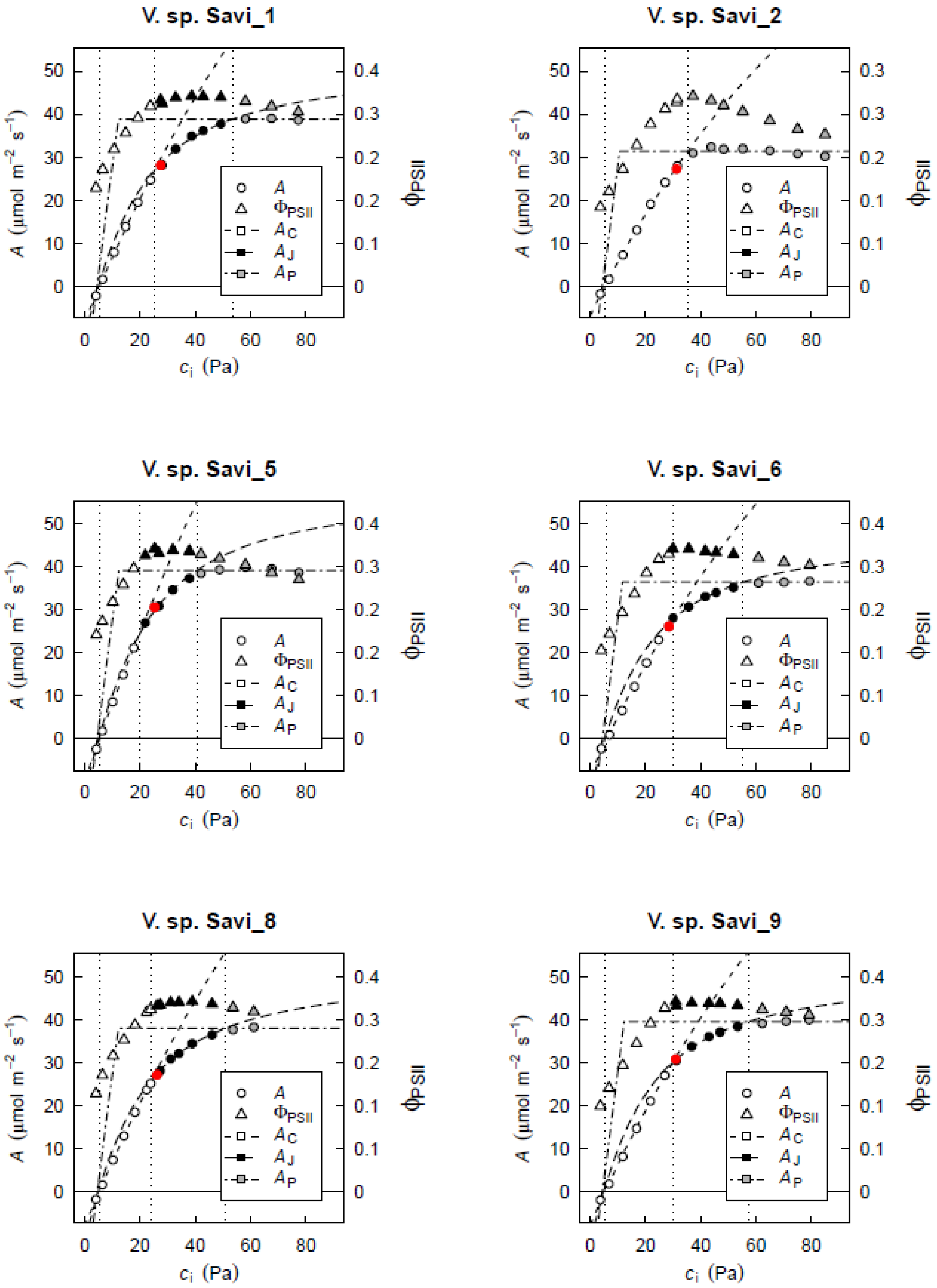

Fig. S7 continued.

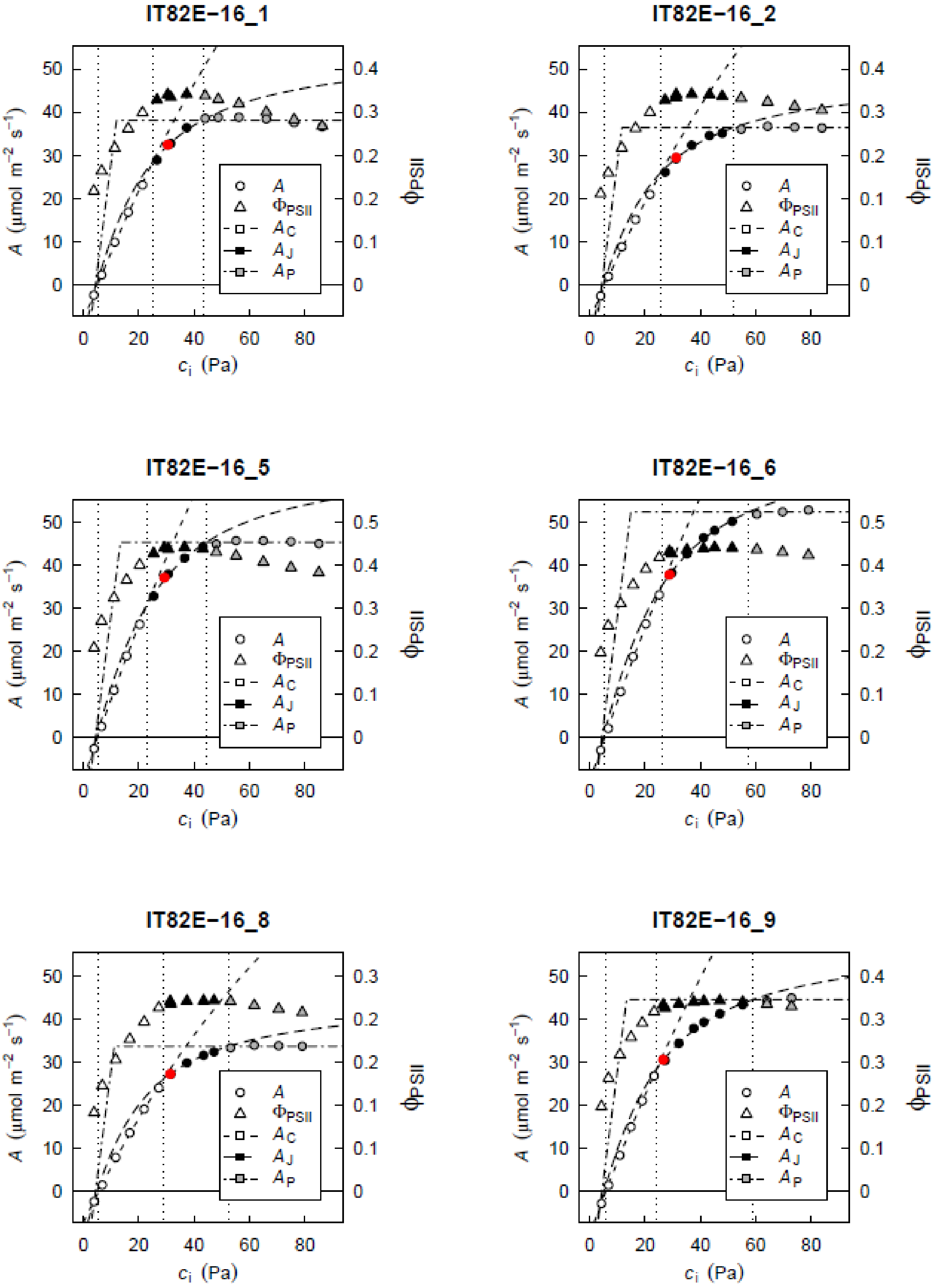

Fig. S7 continued.

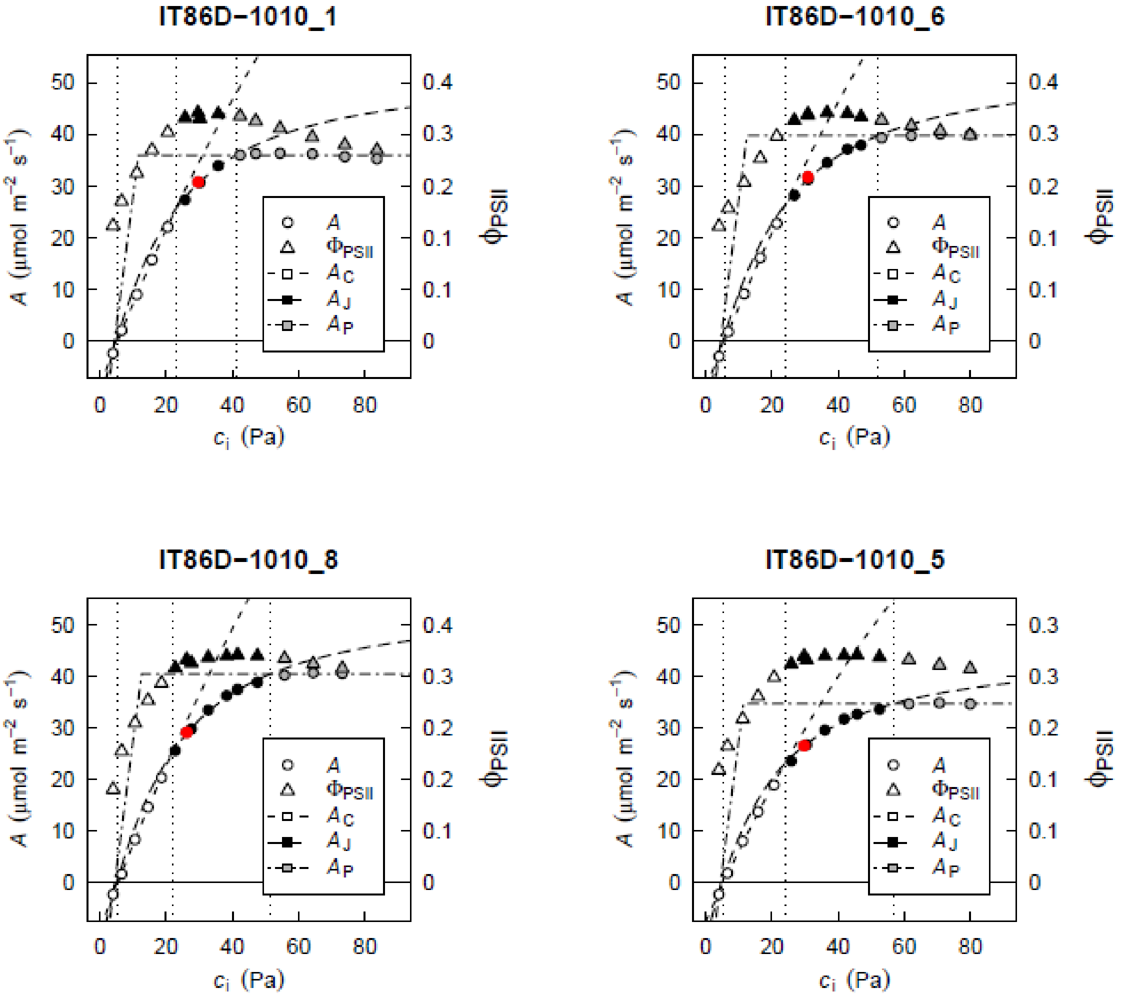

Fig. S8. Duration of Rubisco limitation

The duration of Rubisco limitation was assessed based on intersection between net CO_2 assimilation (A, black line) and potential RuBP-regeneration limited net CO_2 assimilation predicted from c_i using parameters from steady-state A/c_i responses (A_J, red line). Transparent lines show complete timeseries; overplotted solid lines show segments used in modelling (Fig. S10). Vertical dashed lines indicate 5 min induction. Individuals coded as in Table S3; A_J is not provided for *V.* sp. Savi_2, consistent with its steady state A/c_i (Fig. S7).

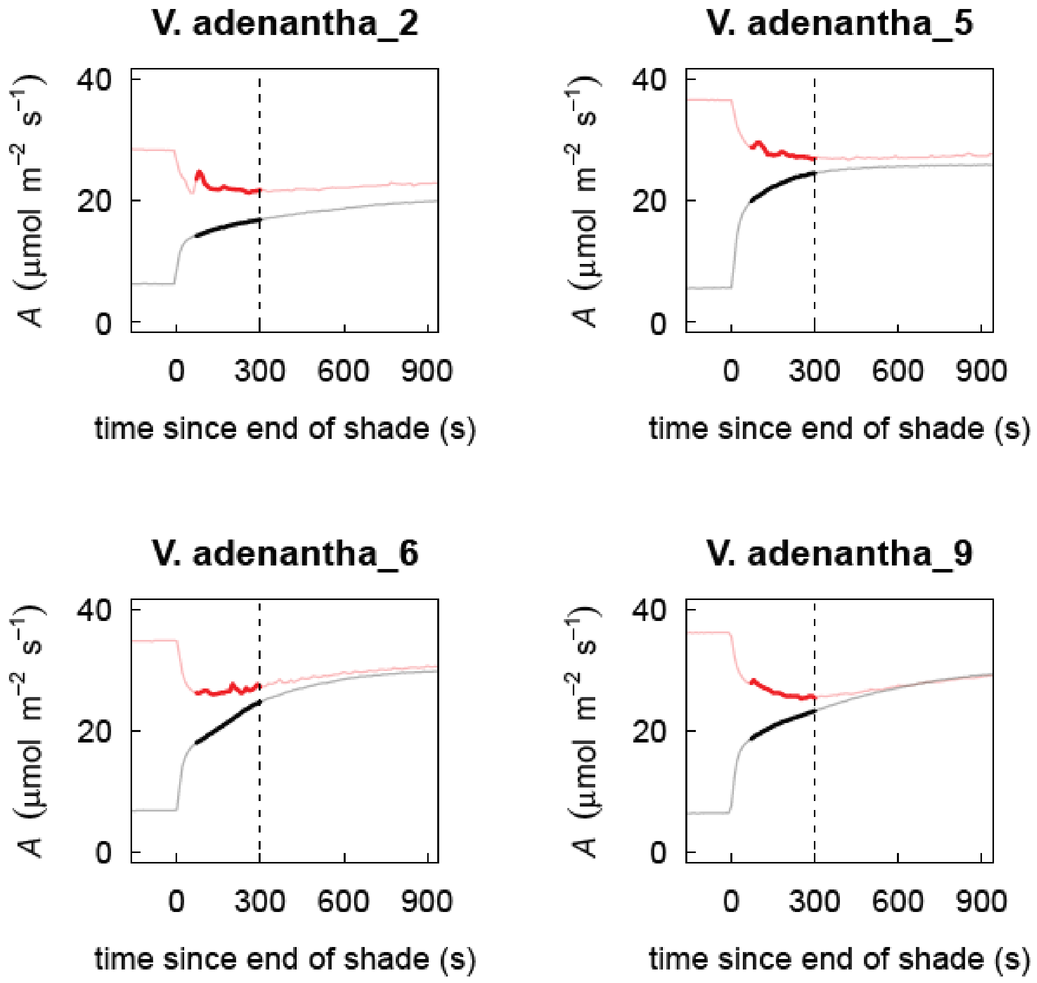

Fig. S8 continued.

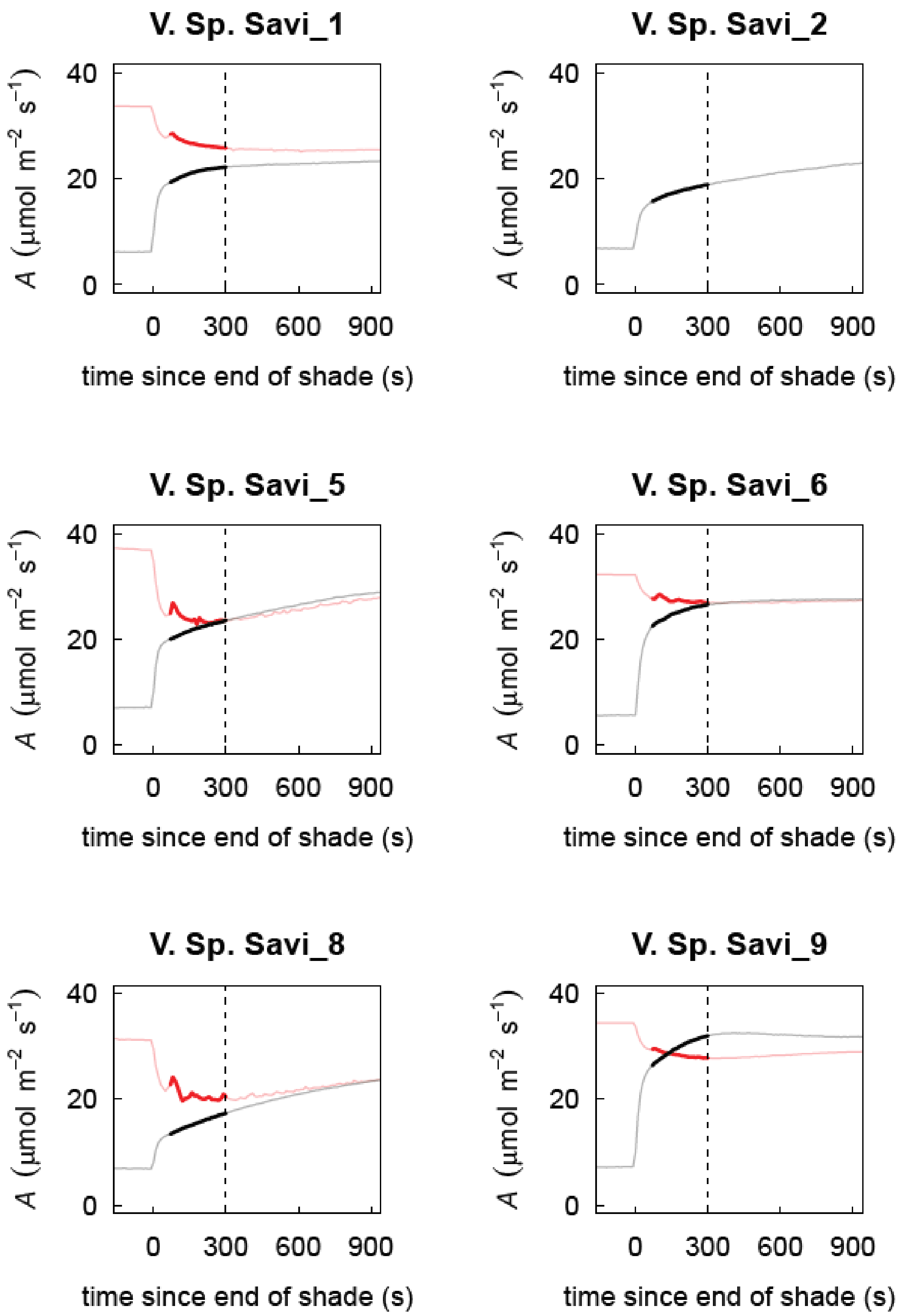

Fig. S8 continued.

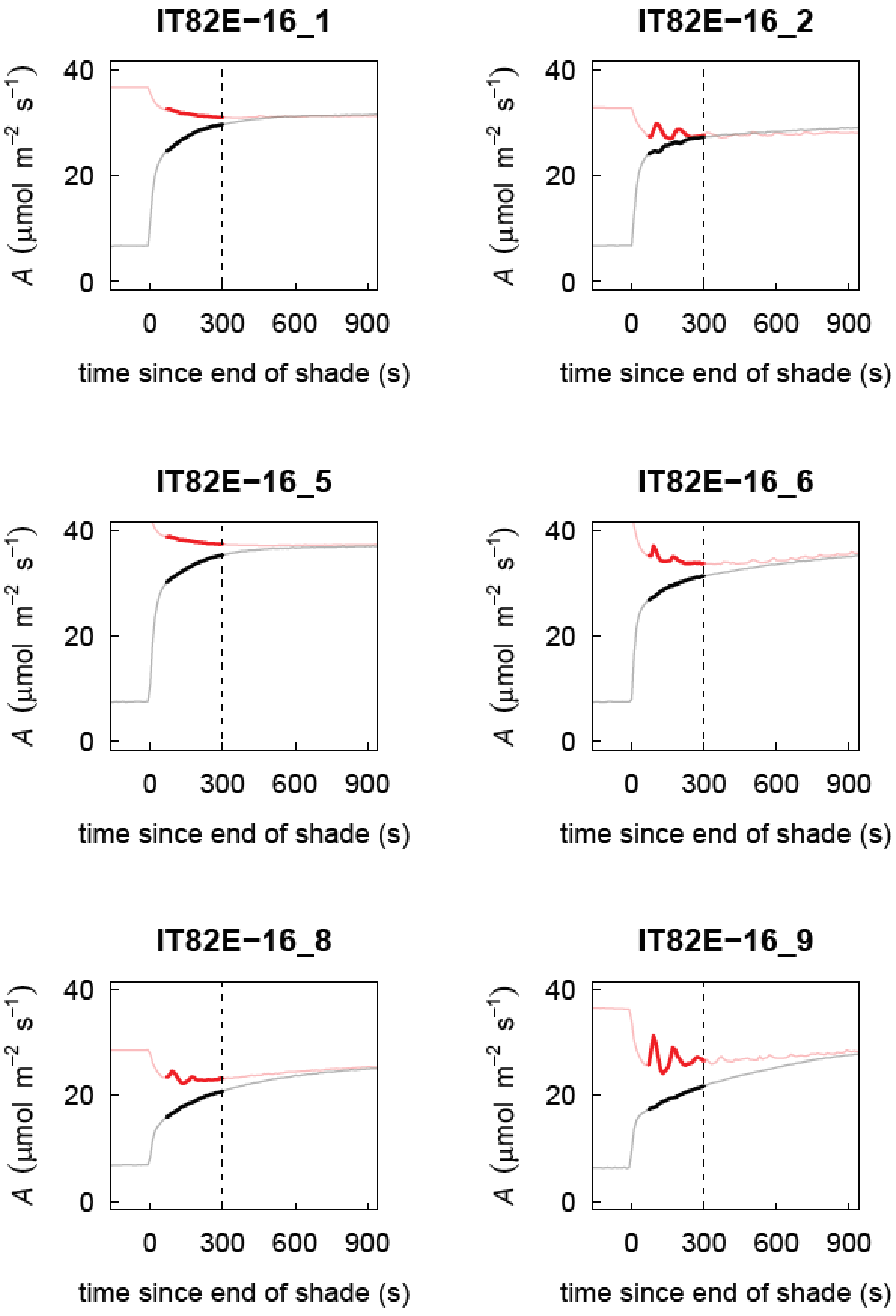

Fig. S8 continued.

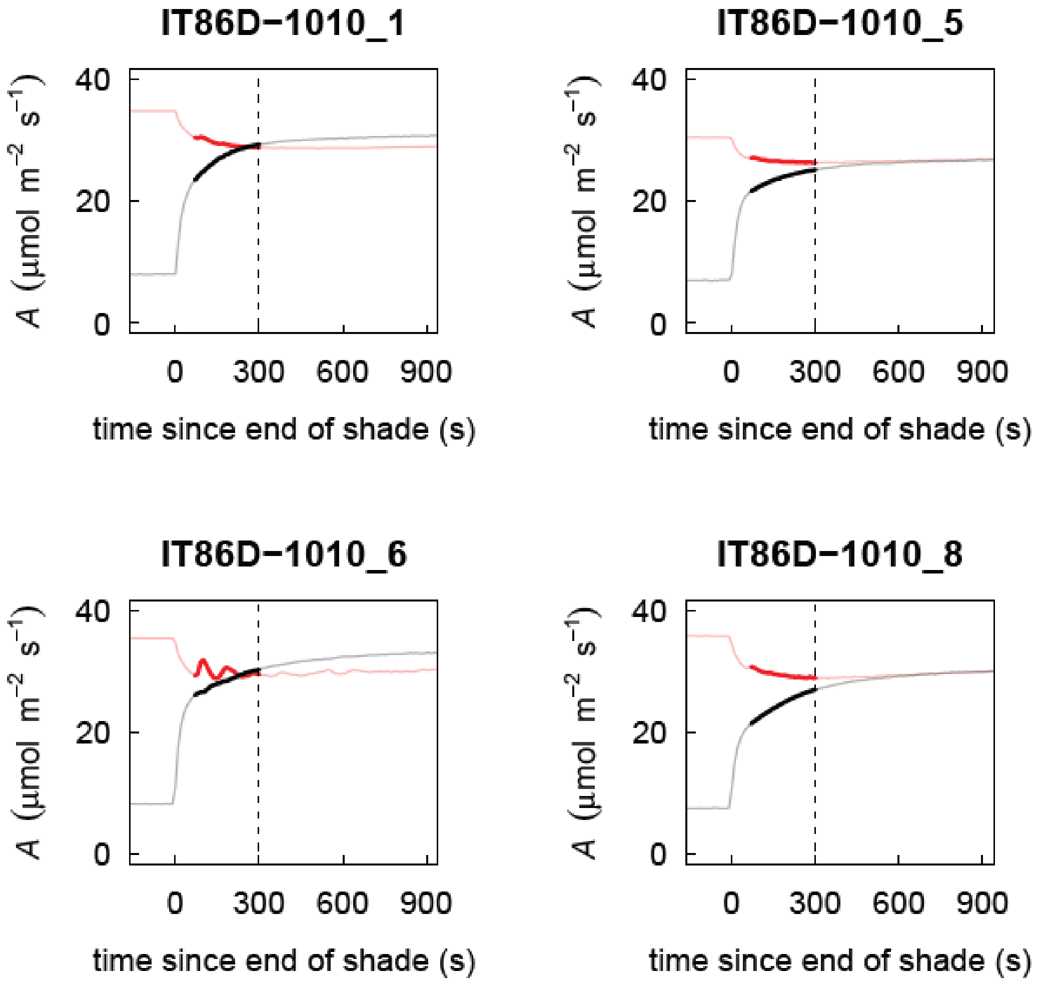

Fig. S9. Sun-shade-sun responses for Rubisco activation state (*S*)
Individual replicates are plotted, with overlaid best-fit models at the levels of: genotypes, solid lines; individuals, dashed lines. Individuals coded as in Table S2.

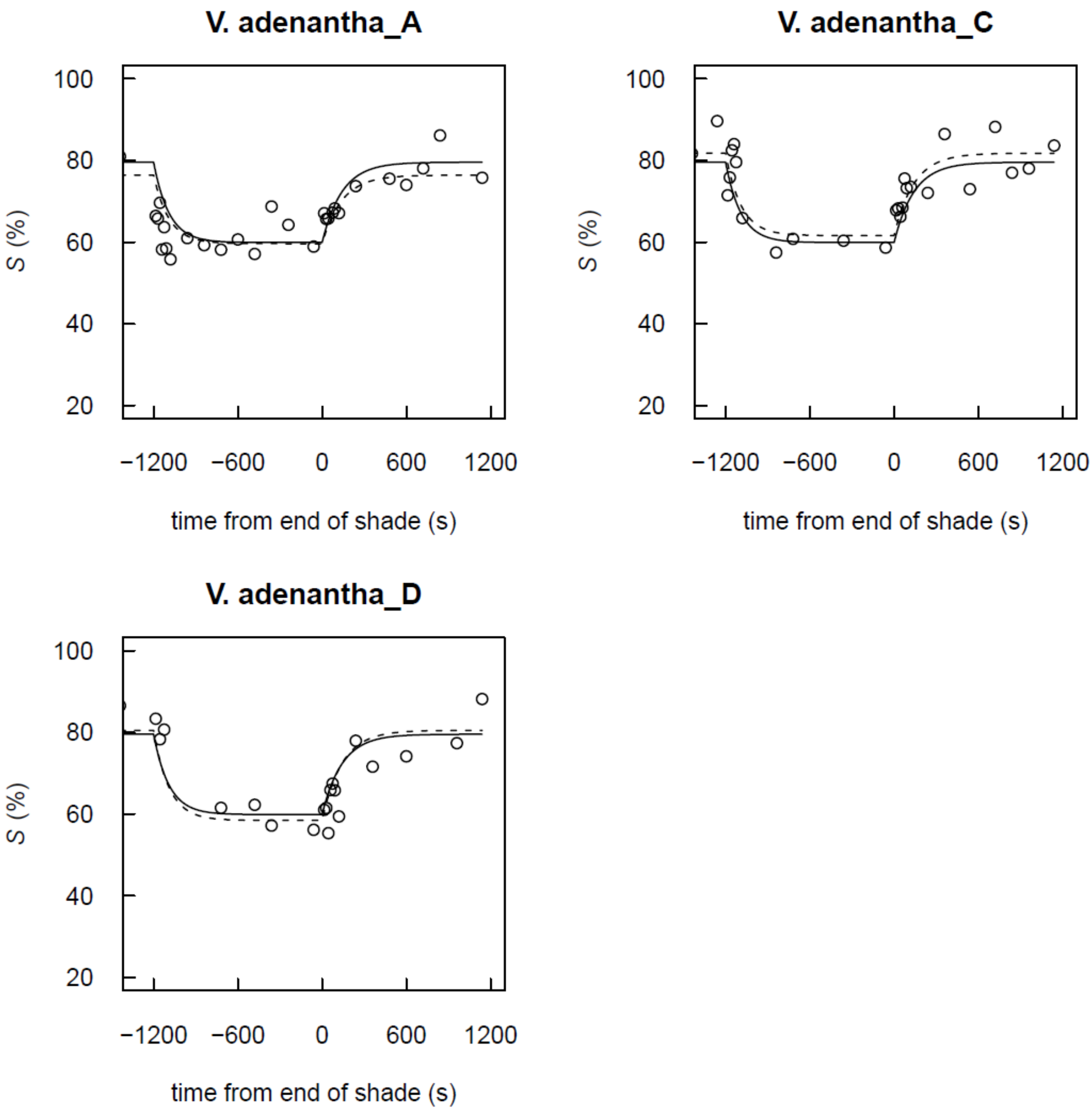

Fig. S9 continued.

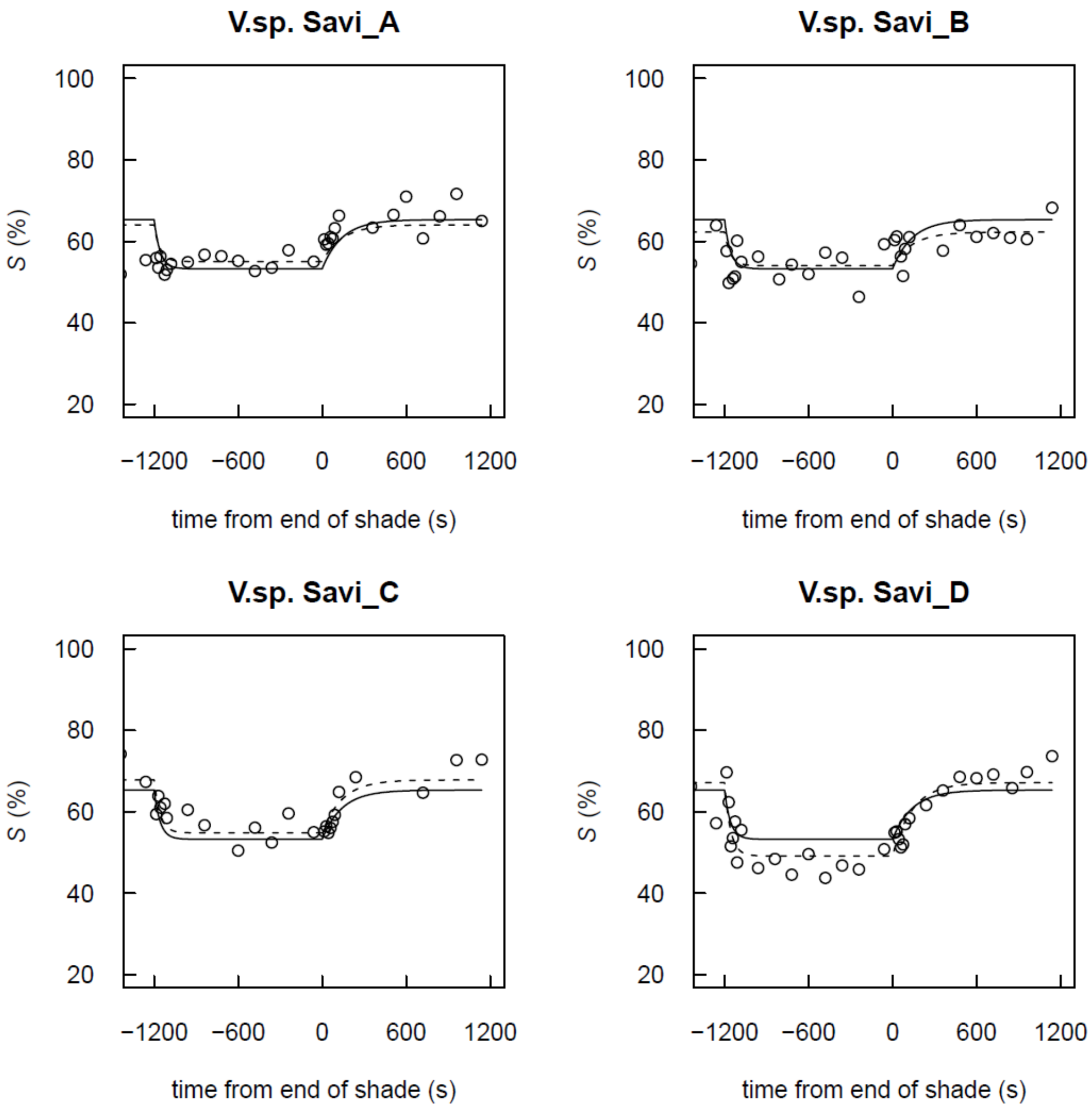

Fig. S9 continued.

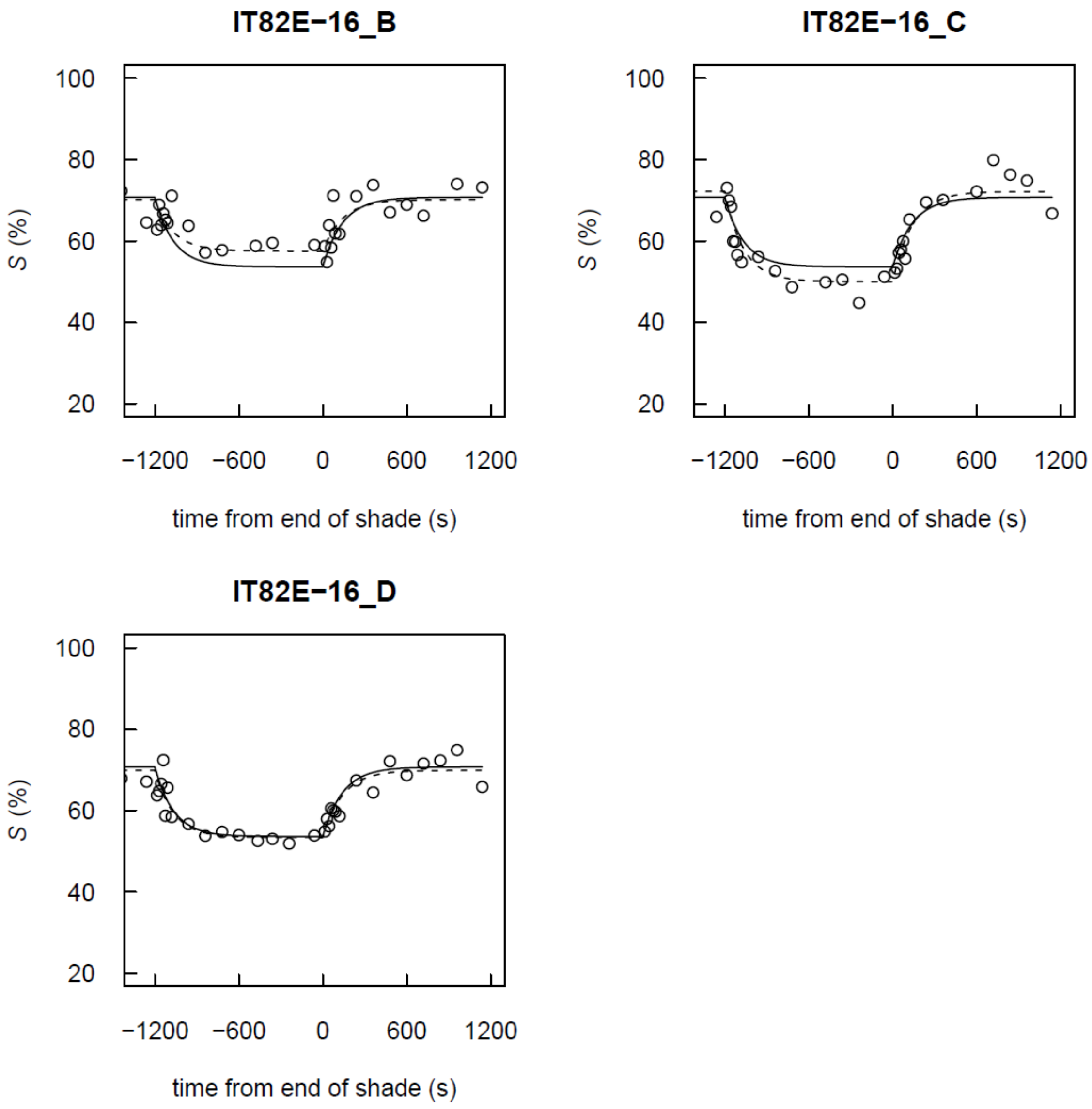

Fig. S9 continued.

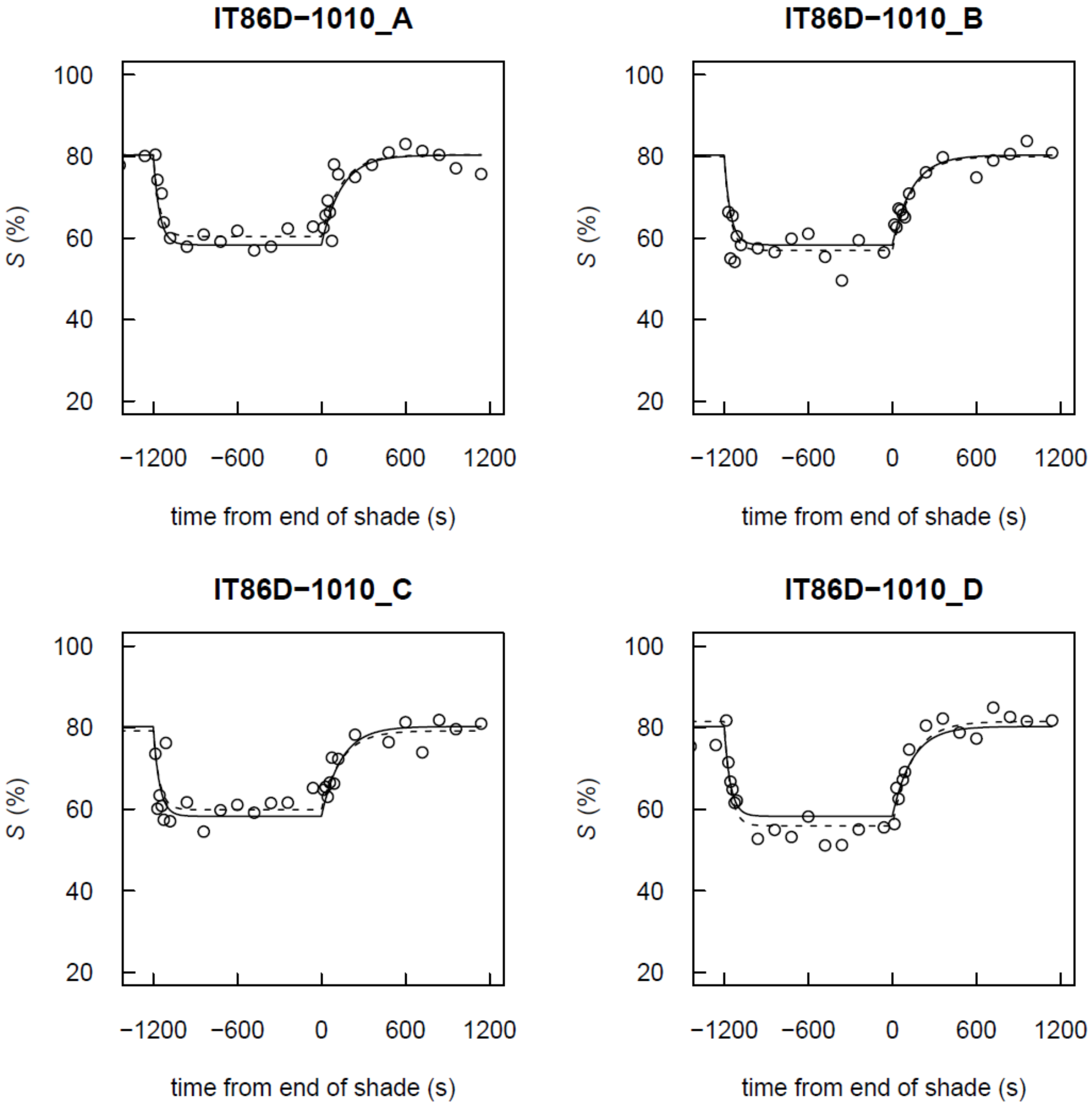

Fig. S10. Induction (shade-sun) responses for one-point $V_{c,max}$

Individual replicates are plotted, with overlaid best-fit models at the levels of: genotypes, solid lines; individuals, dashed lines. Individuals coded as in Table S2. Extrapolations show predictions from the end of shade through to 10 min induction.

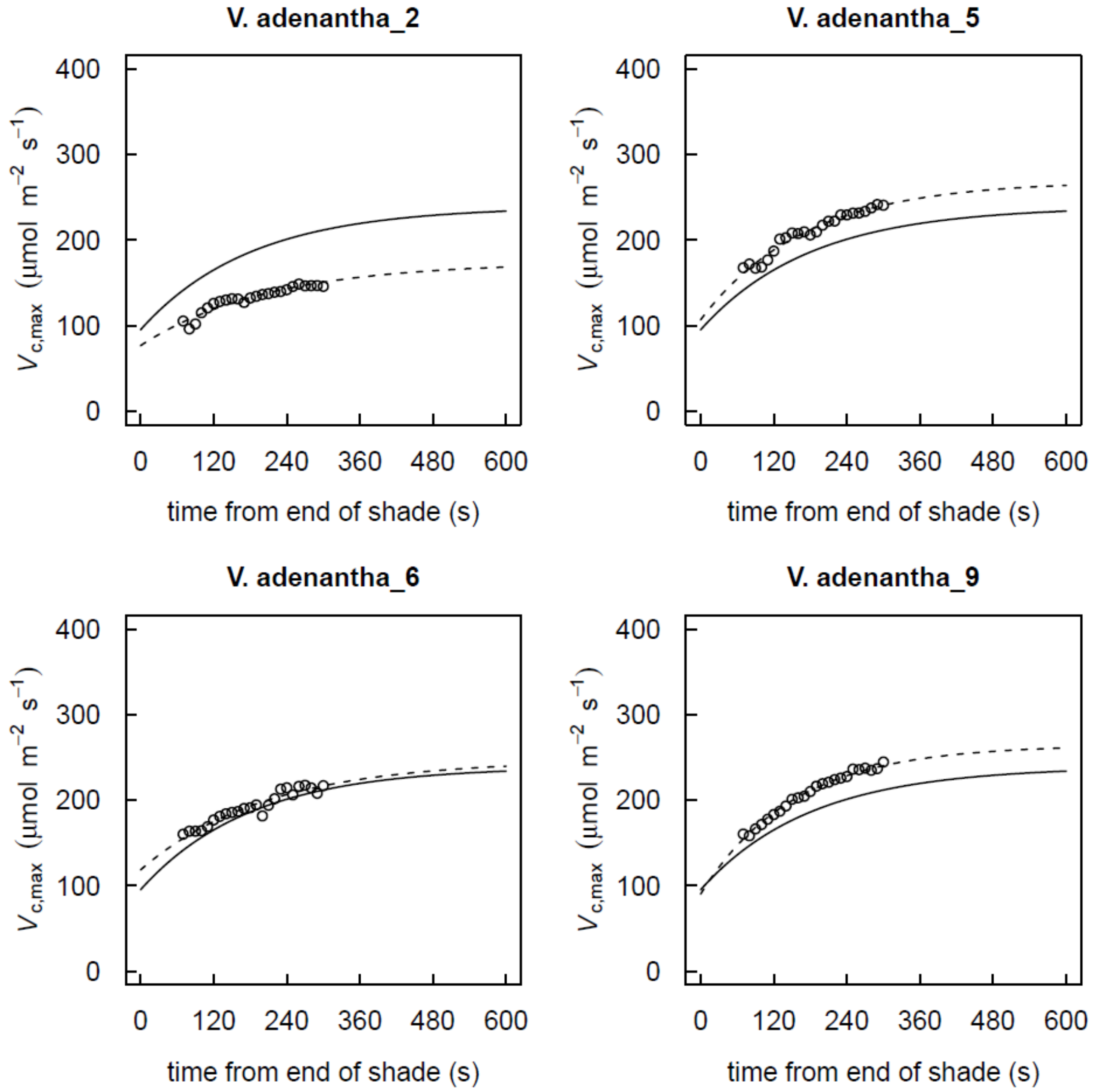

Fig. S10 continued.

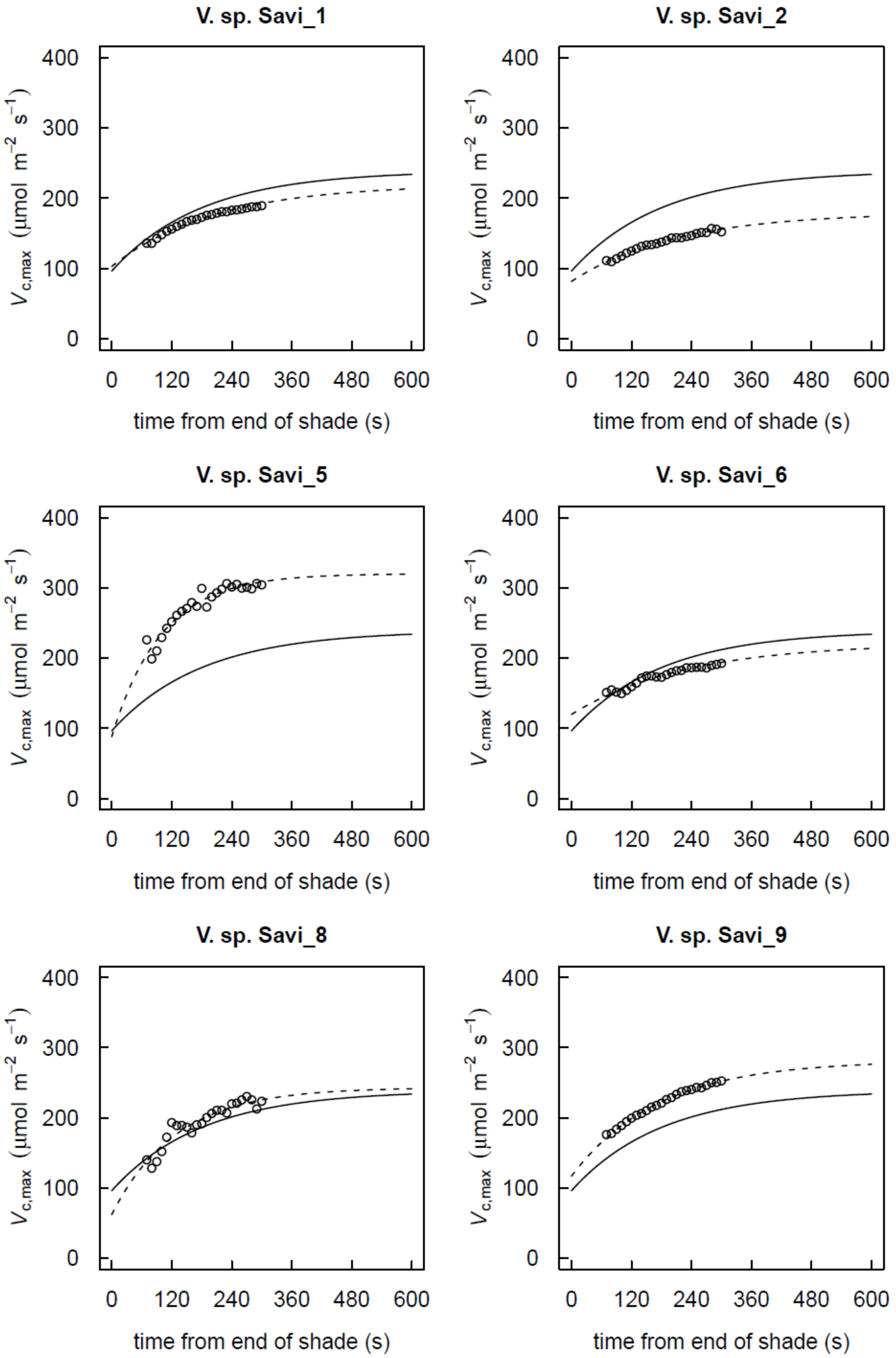

Fig. S10 continued.

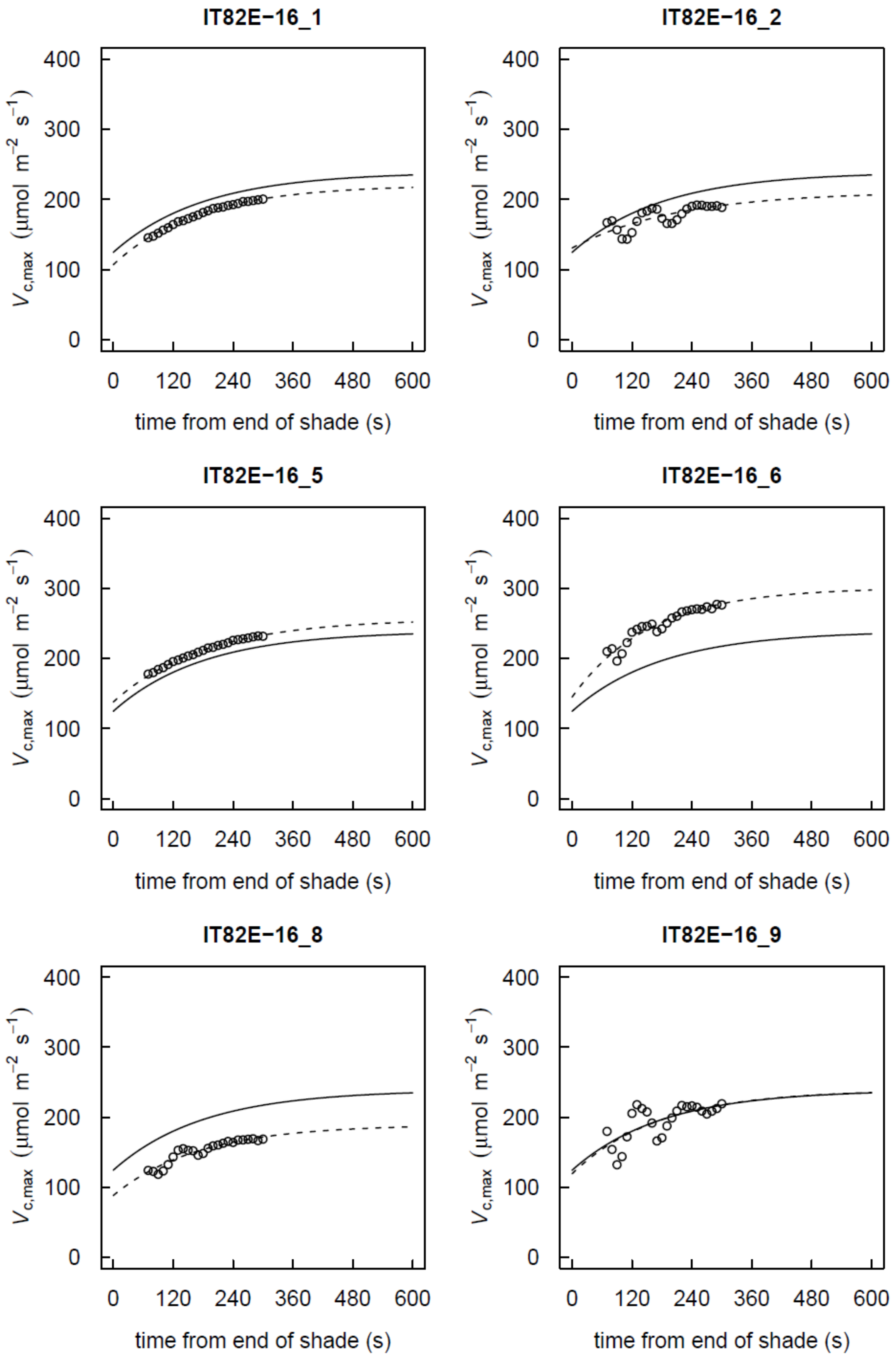

Fig. S10 continued.

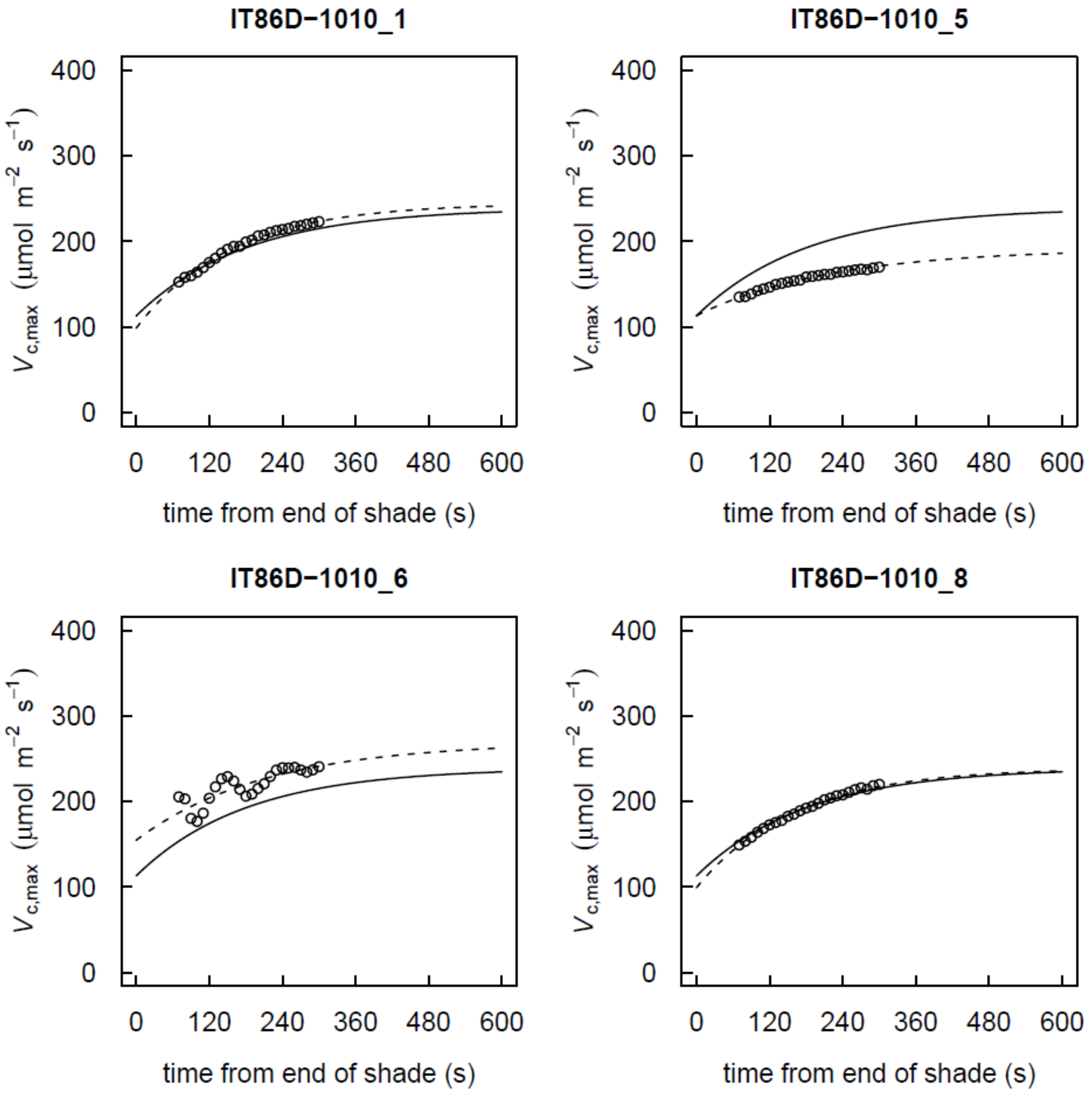

Fig. S11. A/PPFD responses

Individual replicates are plotted, with overlaid best-fit models at the levels of: genotypes, solid lines; individuals, dashed lines. Individuals coded as in Table S3.

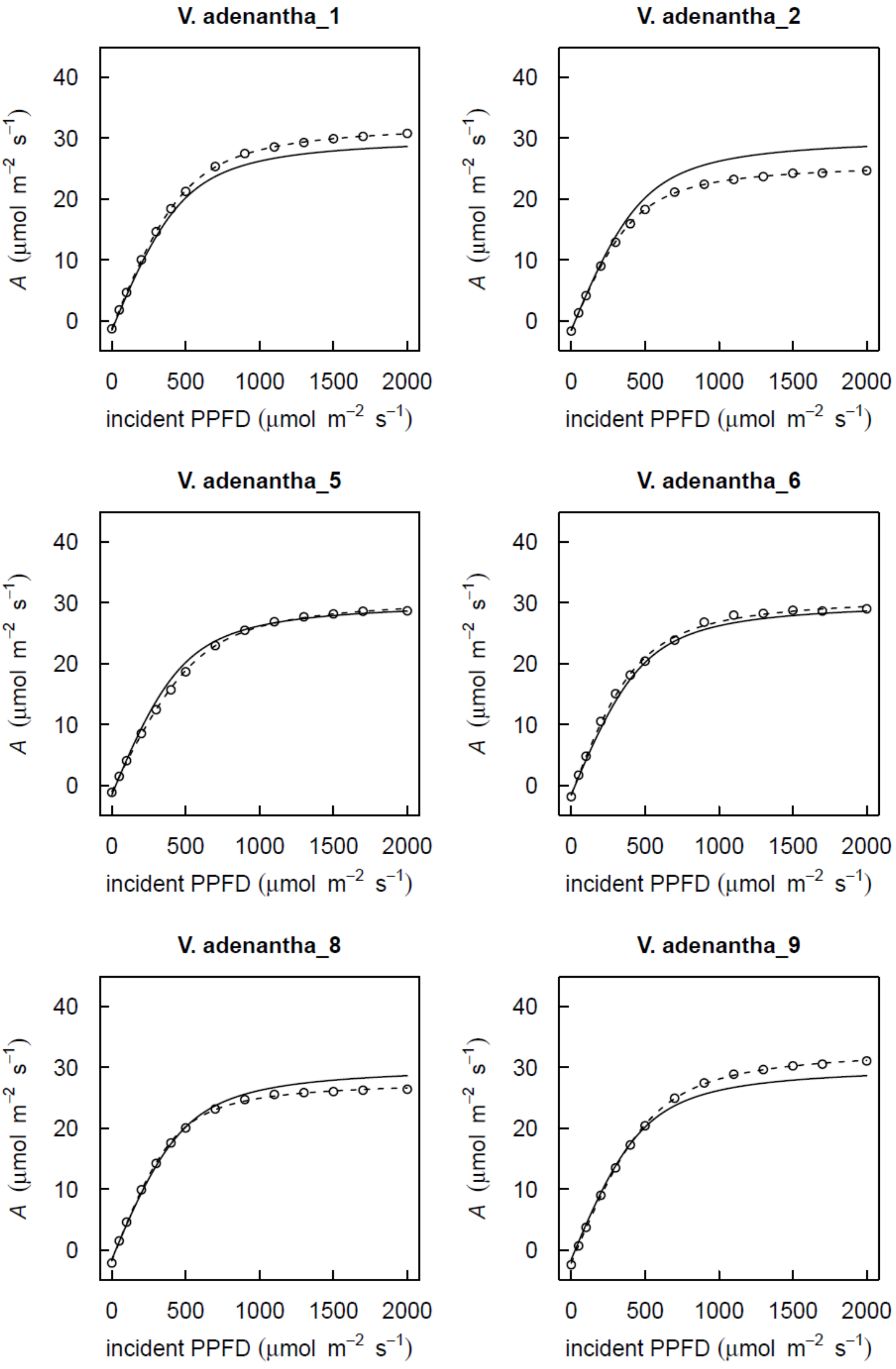

Fig. S11 continued.

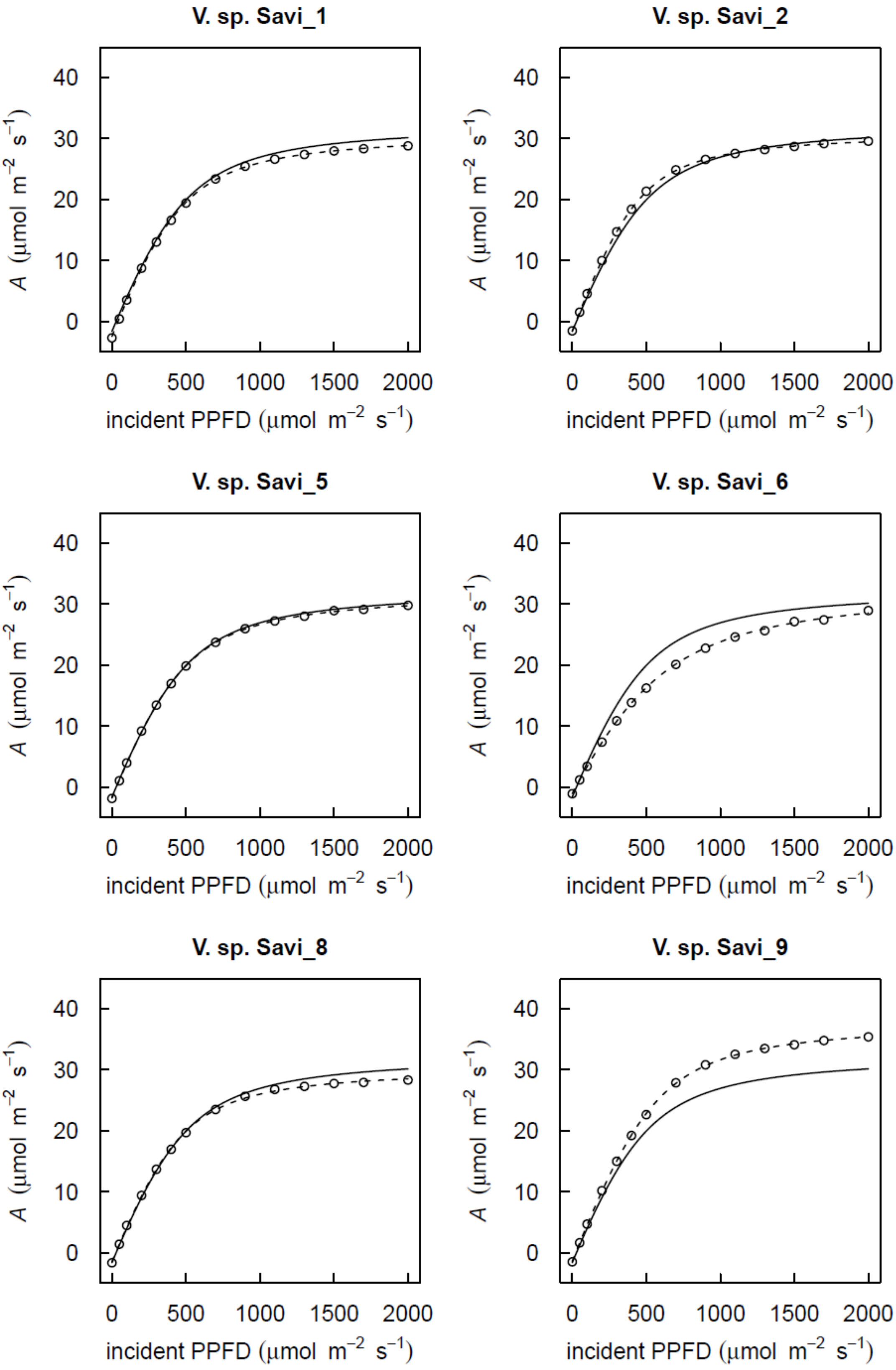

Fig. S11 continued.

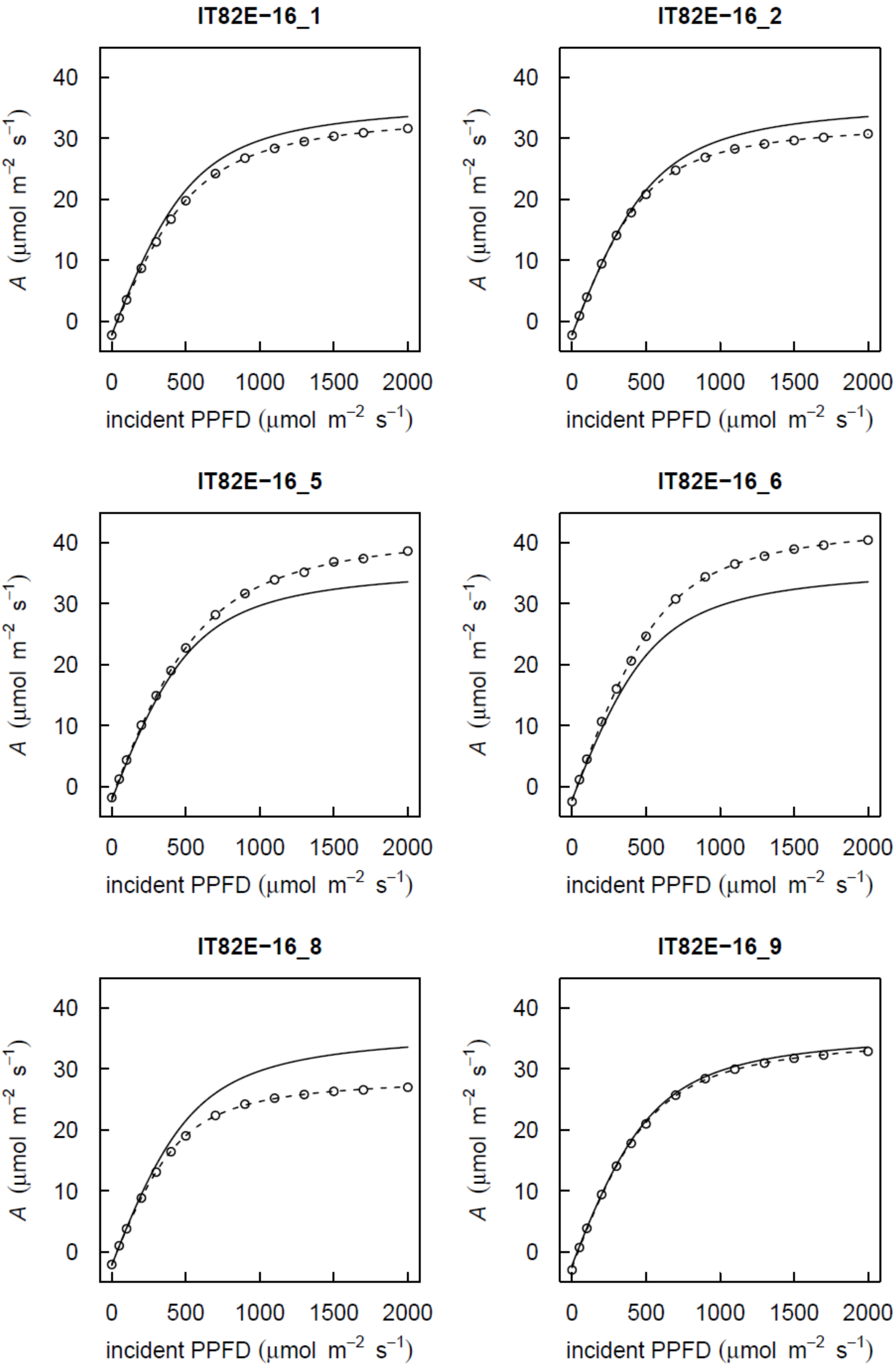

Fig. S11 continued.

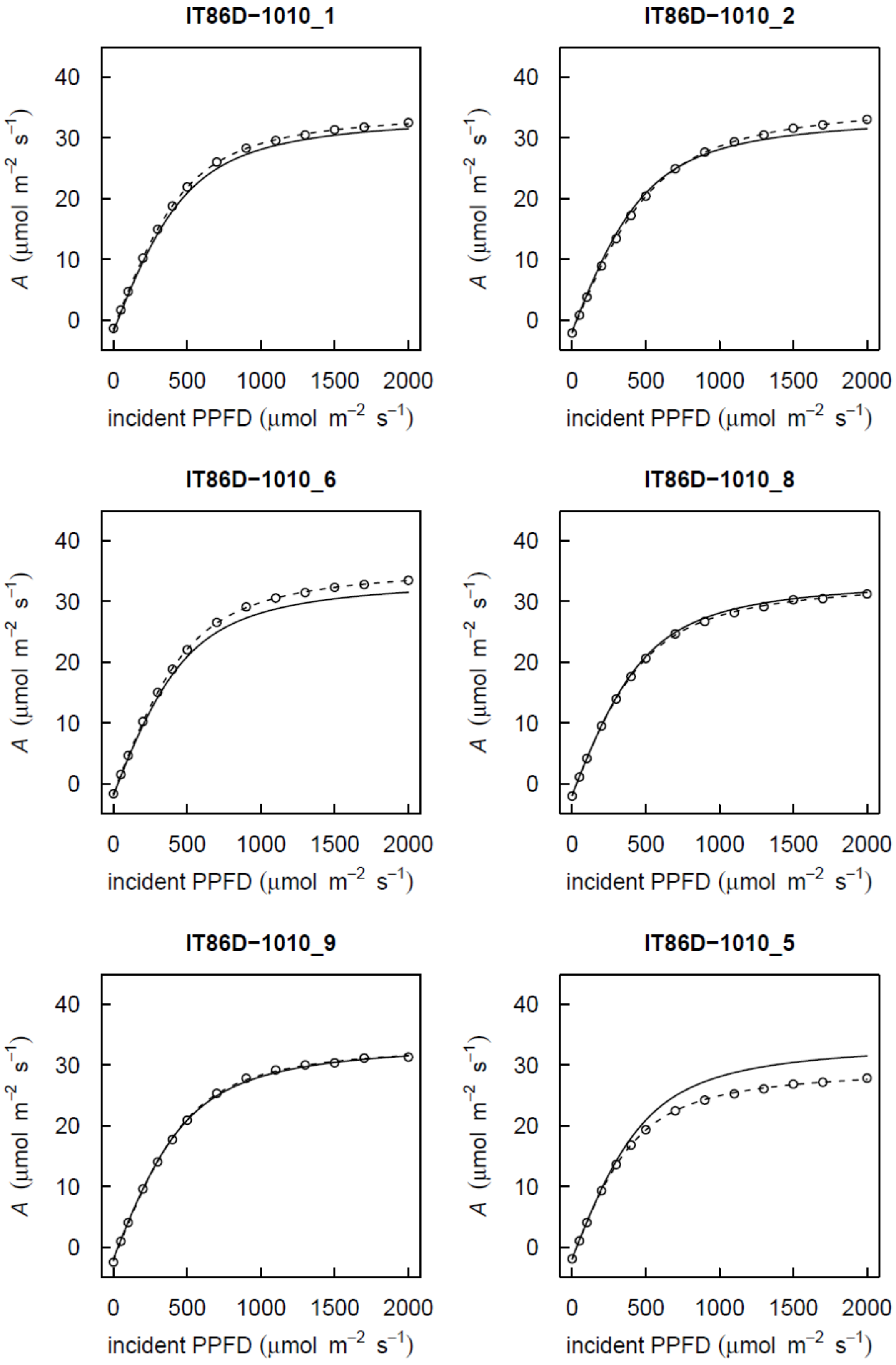

References

1 Long, S. P. & Hallgren, J.-E. Measurement of CO_2 assimilation by plants in the field and the laboratory. in *Photosynthesis and Production in a Changing Environment: a field and laboratory manual* (eds. Hall, D. O., Scurlock, J. M. O., Bolhàr-Nordenkampf, H. R., Leegood, R. C. & Long, S. P.) 62–94, doi:10.1016/B978-0-08-031999-5.50016-9 (1993).

2 Marshall, B. & Biscoe, P. V. A model for C_3 leaves describing the dependence of net photosynthesis on irradiance. *J. Exp. Bot.* **31**, 29–39 (1980).

3 R Core Team. R: A language and environment for statistical computing. R Foundation for Statistical Computing, Vienna, Austria. URL https://www.R-project.org/. (2020).

4 Von Caemmerer, S. & Farquhar, G. D. Some relationships between the biochemistry of photosynthesis and the gas exchange of leaves. *Planta* **153**, 376–387 (1981).

5 Farquhar, G. D., von Caemmerer, S. & Berry, J. A. A Biochemical Model of Photosynthetic CO_2 Assimilation in Leaves of C_3 Species. *Planta* **149**, 78–90 (1980).

6 Gu, L., Pallardy, S. G., Tu, K., Law, B. E. & Wullschleger, S. D. Reliable estimation of biochemical parameters from C_3 leaf photosynthesis-intercellular carbon dioxide response curves. *Plant, Cell Environ.* **33**, 1852–1874 (2010).

7 Taylor, S. H., Orr, D. J., Carmo-Silva, E. & Long, S. P. During photosynthetic induction, biochemical and stomatal limitations differ between Brassica crops. *Plant Cell Environ.* **43**, 2623–2636, doi:10.1111/pce.13862 (2020).

8 Sharkey, T. D., Bernacchi, C. J., Farquhar, G. D. & Singsaas, E. L. Fitting photosynthetic carbon dioxide response curves for C_3 leaves. *Plant. Cell Environ.* **30**, 1035–40 (2007).

9 Busch, F. A. & Sage, R. F. The sensitivity of photosynthesis to O_2 and CO_2 concentration identifies strong Rubisco control above the thermal optimum. *New Phytol.* **213**, 1036–1051 (2017).
