# Supplementary material for: Faster than expected Rubisco deactivation in shade reduces cowpea photosynthetic potential in variable light conditions
Source: Nat Plants. 2022 Jan 20;8(2):118–24. doi: 10.1038/s41477-021-01068-9 (PMC8863576; doi:10.1038/s41477-021-01068-9)
Supplement: Supplementary file 2 — Reporting Summary [file 41477_2021_1068_MOESM2_ESM.pdf]

nature research

Corresponding author(s): Elizabete Carmo-Silva

Last updated by author(s): Nov 10, 2021

Reporting Summary

Nature Research wishes to improve the reproducibility of the work that we publish. This form provides structure for consistency and transparency in reporting. For further information on Nature Research policies, see our Editorial Policies and the Editorial Policy Checklist.

Statistics

For all statistical analyses, confirm that the following items are present in the figure legend, table legend, main text, or Methods section.

n/a	Confirmed	
☐	☒	The exact sample size (*n*) for each experimental group/condition, given as a discrete number and unit of measurement
☐	☒	A statement on whether measurements were taken from distinct samples or whether the same sample was measured repeatedly
☐	☒	The statistical test(s) used AND whether they are one- or two-sided<br>*Only common tests should be described solely by name; describe more complex techniques in the Methods section.*
☒	☐	A description of all covariates tested
☐	☒	A description of any assumptions or corrections, such as tests of normality and adjustment for multiple comparisons
☐	☒	A full description of the statistical parameters including central tendency (e.g. means) or other basic estimates (e.g. regression coefficient) AND variation (e.g. standard deviation) or associated estimates of uncertainty (e.g. confidence intervals)
☐	☒	For null hypothesis testing, the test statistic (e.g. F, t, r) with confidence intervals, effect sizes, degrees of freedom and P value noted<br>*Give P values as exact values whenever suitable.*
☒	☐	For Bayesian analysis, information on the choice of priors and Markov chain Monte Carlo settings
☒	☐	For hierarchical and complex designs, identification of the appropriate level for tests and full reporting of outcomes
☒	☐	Estimates of effect sizes (e.g. Cohen's *d*, Pearson's *r*), indicating how they were calculated

Our web collection on statistics for biologists contains articles on many of the points above.

Software and code

Policy information about availability of computer code

Data collection	Photosynthetic gas-exchange data was collected using LI-6800F Photosynthesis Systems and the associated software (Bluestem v.1.2.2, Scripts version 2017.12 1.2.1, Oct 2017, and Fluorometer version 1.1.6), which are commercially available (LI-COR Inc., Lincoln NE, USA).
Data analysis	Modelling and statistical analyses were implemented in R Language and Environment (v4.1.1), using RStudio (v1.4.1103) with version control. Custom code used to fit A/ci and A/Q responses, alongside the complete analysis workflow, is available via GitHub (https://github.com/smuel-tylor/Fast-Deactivation-of-Rubisco; CC0 licence).

For manuscripts utilizing custom algorithms or software that are central to the research but not yet described in published literature, software must be made available to editors and reviewers. We strongly encourage code deposition in a community repository (e.g. GitHub). See the Nature Research guidelines for submitting code & software for further information.

Data

Policy information about availability of data

All manuscripts must include a data availability statement. This statement should provide the following information, where applicable:

- Accession codes, unique identifiers, or web links for publicly available datasets
- A list of figures that have associated raw data
- A description of any restrictions on data availability

The data that support the findings of this study are available in the Lancaster University Research Directory: https://doi.org/10.17635/lancaster/researchdata/493. They are also included, for ease of use, with the aforementioned GitHub submission (https://github.com/smuel-tylor/Fast-Deactivation-of-Rubisco)

Field-specific reporting

Please select the one below that is the best fit for your research. If you are not sure, read the appropriate sections before making your selection.

☒ Life sciences ☐ Behavioural & social sciences ☐ Ecological, evolutionary & environmental sciences

For a reference copy of the document with all sections, see nature.com/documents/nr-reporting-summary-flat.pdf

Life sciences study design

All studies must disclose on these points even when the disclosure is negative.

Sample size	The minimum sample size of 3 independent biological replicates was limited by the effort required to obtain each set of measurements. More samples were collected where possible, up to a maximum of n = 6.
Data exclusions	Data that failed to match physiological expectations, were noisily imprecise, or failed to produce adequate fits when carrying out non-linear modelling were excluded. These exclusions and their rationale are documented for the complete gas exchange dataset in data analysis scripts made available on GitHub. Rubisco activity data was quality checked for errors in experimental procedure.
Replication	Biological replicates were analysed independently and all genotypes were measured at the same time. A minimum of 3 and maximum of 6 independent biological replicates were collected in each experiment.
Randomization	Plants were distributed according to a random block design, with every genotype represented in each block and plants of the various genotypes distributed randomly in each block. A minimum of 4 blocks was used per experiment. Samples were processed in random order.
Blinding	There was no blinding. Due to the experiment design, researchers were aware of the identity of each plant because, for example, visual checking for leaf age prior to sampling was a necessary component of the protocols and the genotypes have different leaf shapes. For Rubisco activity experiments, leaf disc samples undergo processing subsequent to sampling; processing was done in batches that ensured all treatments and accessions were treated similarly.

Reporting for specific materials, systems and methods

We require information from authors about some types of materials, experimental systems and methods used in many studies. Here, indicate whether each material, system or method listed is relevant to your study. If you are not sure if a list item applies to your research, read the appropriate section before selecting a response.

Materials & experimental systems

n/a	Involved in the study	
☒	☐	Antibodies
☒	☐	Eukaryotic cell lines
☒	☐	Palaeontology and archaeology
☒	☐	Animals and other organisms
☒	☐	Human research participants
☒	☐	Clinical data
☒	☐	Dual use research of concern

Methods

n/a	Involved in the study	
☒	☐	ChIP-seq
☒	☐	Flow cytometry
☒	☐	MRI-based neuroimaging
